# Degradable poly(β-amino ester) microparticles for cleansing products and food fortification

**DOI:** 10.1038/s44286-024-00151-0

**Published:** 2024-12-06

**Authors:** Linzixuan Zhang, Ruiqing Xiao, Tianyi Jin, Xinyan Pan, Katharina A. Fransen, Shahad K. Alsaiari, Alicia Lau, Ruizhe He, Jooli Han, Benjamin J. Pedretti, Jing Ying Yeo, Xin Yang, Bradley D. Olsen, Alfredo Alexander-Katz, Zachary P. Smith, Robert Langer, Ana Jaklenec

**Affiliations:** 1 Department of Chemical Engineering, Massachusetts Institute of Technology, Cambridge, MA USA; 2 David H. Koch Institute for Integrative Cancer Research, Massachusetts Institute of Technology, Cambridge, MA USA; 3 Department of Biological Engineering, Massachusetts Institute of Technology, Cambridge, MA USA; 4 Department of Materials Science and Engineering, Massachusetts Institute of Technology, Cambridge, MA USA

**Keywords:** Chemical engineering, Polymers, Polymer synthesis

## Abstract

Microplastic pollution is a pressing global crisis caused by the extensive use of nondegradable microplastic materials in daily activities. One effective approach to mitigate this issue is to replace nondegradable plastics with degradable materials that have properties amendable for targeted applications. Here we present the development of a degradable microparticle (MP) platform based on a poly(β-amino ester) (PAE) that degrades into sugar and amino acid derivatives. This PAE MP platform showed functional replacement of nondegradable microplastics used in cleansing products and food fortification. In cleansing products, PAE MPs effectively enhanced the cleansing efficiency of a representative rinse-off product and showed effective removal of potentially toxic elements, as an alternative of traditional nondegradable microbeads. In food fortification, PAE MPs provided robust protection for multiple essential vitamins and minerals against extensive cooking and storage conditions with rapid nutrient release in a simulated human digestion system. Collectively, these PAE MPs present a potential platform to replace microplastic usage on a global scale in many applications.

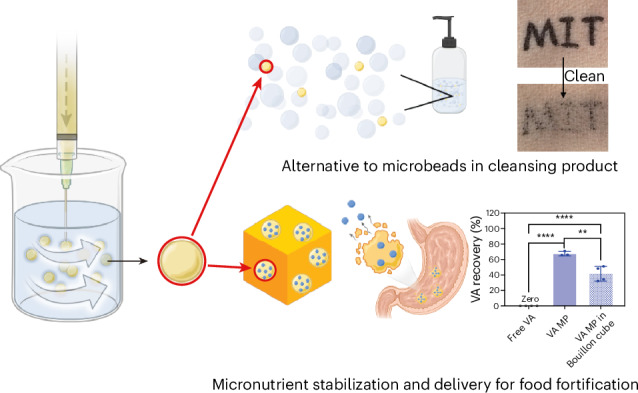

## Abstract (web summary)

This study reports the preparation of degradable poly(β-amino ester) microparticles as a promising replacement for nondegradable microplastics in cleansing products and food fortification, demonstrating effective cleansing, toxic element removal and robust nutrient protection with efficient release.

## Main

Microplastic pollution is a growing global environmental crisis. Recent estimates suggest that 500,000 to 8 million metric tons of plastic are discharged into the ocean annually, with a 4% growth rate, where microplastics account for 49–53 kilotonnes^1,2^. Microplastics, defined as synthetic solid particles ranging from 1 μm to 5 mm, resist degradation and are ubiquitous across the food chain, posing environmental and health risks^3–5^. Studies indicate that patients with microplastics in carotid artery plaque face increased risks of heart attack, stroke or death^6^. Microplastics can also carry toxic chemicals into the bloodstream, presenting additional health concerns^7^. Consequently, microplastics have been banned in rinse-off consumer products in the United States and other regions, with the European Commission also proposing stricter regulations^8,9^.

Mitigation strategies include micrometer-scale filtration in wastewater treatment, which has seen limited success owing to infrastructure challenges, and catalytic transformations of plastic waste into other products^10,11^. However, replacing nondegradable microplastics with degradable alternatives provides a potentially more sustainable approach^12^. In this study, we evaluated the potential of a degradable polymeric microparticle (MP) platform as an alternative to nondegradable microplastics in two applications: (1) microbead additives in personal care products and (2) encapsulants for oral delivery of micronutrients for food fortification. Both applications rely on microscale polymer particles, mostly fabricated from nondegradable materials. MPs are used as microbeads in personal care products and as stabilizers for nutrient release in food fortification^13,14^. These applications provide a proof of concept for a versatile MP platform to replace microplastics.

Although degradable polymers have been explored extensively, implementation as replacements for microplastics remains challenging^15,16^. Microplastics are often intentionally added to consumer products, contributing substantially to microplastic discharge^17^. Replacing these materials with degradable polymers requires meeting several criteria: safety for topical and oral use, versatile material properties (for example, tunable hydrophobicity and pH sensitivity), scalability and cost-effectiveness. The polymer must also degrade efficiently under biotic or abiotic factors. Synthetic polymers offer advantages in scalability, consistency and versatility over natural polymers^18^. By altering monomer composition, properties like thermal stability and hydrophobicity can be fine-tuned to meet specific application needs, such as rapid nutrient release in the digestive system for oral delivery^19^.

Previous studies have explored various degradable polymers as alternatives to nondegradable plastics^12,15,20^. However, success has been limited by the diverse material requirements for different applications. To address these needs, we designed a series of natural product-inspired poly(β-amino ester) (PAE) polymers, which are degradable via hydrolysis and offer extensive monomer variation^21,22^. PAE polymers can be synthesized through a step-economical process, providing a practical basis for large-scale fabrication^23^. We formulated PAE MPs with controlled physical properties and demonstrated their successful application as alternatives to microplastics in personal care products and food fortification. The degradability of these MPs into small sugar and amino acid derivatives further highlights their potential environmentally favorable properties.

## Fabrication of MPs from natural product-based polymers

**Fig. 1:**
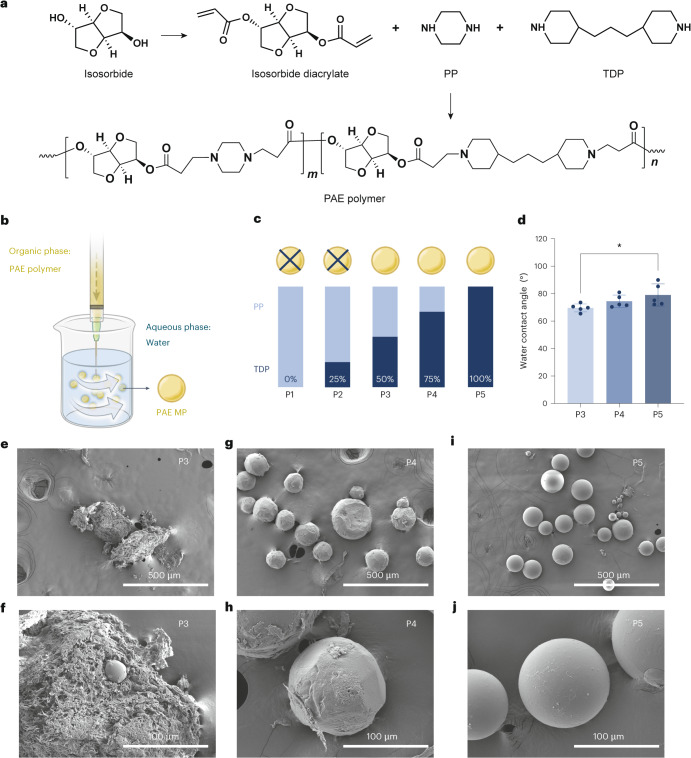
Polymer synthesis and MP fabrication with PAE. **a**, Schemes for the synthesis of PAE with piperazine (PP), TDP and isosorbide diacrylate. **b**, A schematic depiction of the PAE MP fabrication using a modified emulsion method. **c**, Five ratios of PP and TDP were used for PAE synthesis. P3 to P5 compositions successfully formed solid MPs, whereas P1 and P2 did not. **d**, Increasing hydrophobicity for P3, P4 and P5 polymers as demonstrated by water contact angle measurement (*n* = 5 independent replicates; P3 versus P5, *P* = 0.040). **e**–**j**, SEM images showing the surface morphology of PAE MPs formed by P3 (**e** (low magnification) and **f** (high magnification)), P4 (**g** (low magnification) and **h** (high magnification)) and P5 (**i** (low magnification) and **j** (high magnification)) polymers. Data are presented as mean ± s.d. Statistical significance was evaluated using two-tailed Student’s *t*-test. *P* ≤ 0.05 is statistically significant, with *P ≤ 0.05, **P ≤ 0.01, ***P ≤ 0.001 and ****P ≤ 0.0001. Source data

We selected three bifunctional monomers, piperazine, 4,4′-trimethylenedipiperidine (TDP) and a diacrylate-functionalized isosorbide, to construct the PAE polymer scaffold^22^ (Fig. 1a). By varying the feed ratio of piperazine and TDP, we synthesized a group of five polymer compositions: poly[(isosorbide diacrylate)-*co*-piperazine], poly[(isosorbide diacrylate)-*co*-TDP] and copolymers with piperazine and TDP. Each polymer composition was assessed for its compatibility with a modified emulsion-based MP fabrication method^14^. Isosorbide is an advantageous monomer in polymer synthesis owing to its biodegradability and reduced environmental impact, given its biobased origin derived from renewable resources^24^. Its bicyclic structure enhances the rigidity and thermal properties of polymers, which can result in materials with high glass transition temperatures and improved mechanical strength^25^. In addition, isosorbide has been demonstrated as nontoxic with ideal safety and biocompatibility profiles for consumer product applications^26^. Piperazine and TDP were selected owing to their conventional role as monomers in PAE synthesis and their commercial availability^22^. As bis-secondary amines, piperazine and TDP facilitate the formation of polymers with tertiary amines in their backbones without requiring protection and deprotection steps. In addition, the combination of piperazine and TDP allows straightforward tuning of the hydrophobicity of the synthesized polymer. In brief, the PAE polymer was dissolved in dichloromethane that was then dispersed into an aqueous phase under stirring conditions (Fig. 1b). Ranked by the increasing ratio of TDP and piperazine, we labeled the compositions as P1 to P5. Notably, P3, P4 and P5 successfully formed solid, collectable MP-structure materials, whereas P1 and P2 did not and therefore were excluded from further studies (Fig. 1c and Supplementary Fig. 1). To work as an alternative material for microbeads in personal care products, preservation of the spherical structure and smooth surface is needed for mechanical exfoliation and abrasion performances^12^. For food fortification applications, good structural integrity of MPs enhances stabilization efficacy for nutrients and food additives. Previous studies have also shown that sugar-derived chemicals provided a stabilizing effect to micronutrients^27,28^.

The combination of piperazine and TDP as monomers to construct the PAE polymer backbone allowed us to tune the material hydrophobicity effectively. The TDP monomer has a logP of 1.78, while piperazine has a logP of −0.76, resulting in increased hydrophobicity with higher TDP ratios. The P5 polymer (highest TDP ratio) demonstrated the greatest hydrophobicity, as confirmed by water contact angle measurements (Fig. 1d and Supplementary Fig. 2). This enhanced hydrophobicity afforded optimal MP surface morphology for P5 compared with P3 and P4, as characterized by scanning electron microscopy (SEM) imaging (Fig. 1e–j and Supplementary Fig. 3). The P3-based MPs were amorphous with rough surfaces. Interestingly, P4-based MPs showed spherical-like structures with amorphous polymer pieces flocculating on the surface. Fortuitously, P5-based MPs afforded clear spherical structures with smooth surfaces, with size distribution of 76.4 ± 13.3 µm in diameter (Supplementary Fig. 4).

The P5 polymer was singled out for further functional studies given that P5 MPs showed the most optimal spherical shape and smooth surface. These two features are essential for the two select application scenarios. For cleaning products, smooth surfaces with a regular size and shape are considered important criteria for new alternative materials exfoliating use in rinse-off products^12^. In the case of nutrients and food additives, including food fortification, these two features allow MPs to present as free-flowing powders, which are vital for robust encapsulation and compatibility with industrial food processing^29^. Therefore, the homogeneous particle formation with free-flowing properties afforded by P5 MPs presents high potential for easy integration with current food ingredient processing technologies. Such features can potentially accelerate the translation of P5 MPs into real-life applications.

**Fig. 2:**
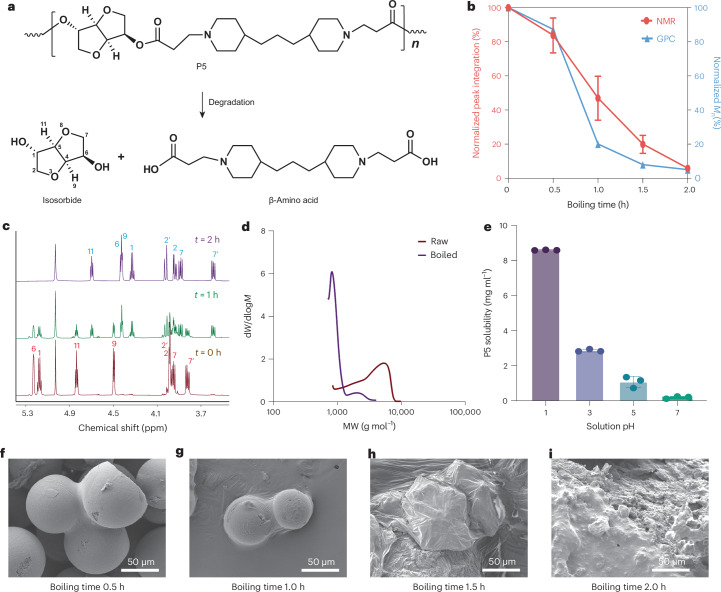
Degradation profile of P5 polymer and MPs. **a**, Scheme of P5 polymer degradation via hydrolysis. **b**, Degradation kinetics of the P5 polymer in boiling water, characterized by NMR and GPC (*n* = 3 independent replicates for NMR). *M*_n_, number-average molecular weight. **c**, NMR spectrum comparison of P5 polymer samples treated with boiling water for various time points, including *t* = 0 h (brown), *t* = 1 h (green) and *t* = 2 h (purple). Peak shifts showed correspondence to the degradation of the polymer, where peak labels are as in panel **a**. **d**, GPC spectrum comparison of untreated and 2 h boiled polymer samples showing substantial molecular weight (MW) change, further confirming polymer degradation. **e**, P5 polymer showed a higher solubility at low pH aqueous solution (*n* = 3 independent replicates). **f**–**i**, P5 MPs were treated with boiling water for 0.5 h (**f**), 1 h (**g**), 1.5 h (**h**) and 2 h (**i**). Particle deformation was observed under SEM imaging. The degree of deformation increased as boiling time progressed. The data are presented as mean ± s.d. Source data

The P5 polymer degrades via hydrolysis into isosorbide and a β-amino acid^22^ (Fig. 2a). Nuclear magnetic resonance (NMR) and gel permeation chromatography (GPC) were used to characterize the degradation kinetics of the P5 polymer MPs in boiling water. Quantitative analysis revealed rapid degradation, with only 5.5% of the P5 polymer remaining after 2 h of exposure (Fig. 2b and Supplementary Fig. 5). NMR spectra from samples at various boiling intervals, including 0 h, 1 h and 2 h, highlighted peak transitions indicative of molecular degradation (Fig. 2c). Integration of peaks 1 and 6 at chemical shift range of 5.1 ppm to 5.3 ppm was used to calculate the degradation rate. Furthermore, GPC spectra comparing the untreated and 2 h boiled samples showed substantial differences in molecular weight, underscoring the extensive degradation under these conditions (Fig. 2d). These results confirm effective degradation of P5 polymer in boiling water. A high-throughput automated biodegradation assay was used to further evaluate P5 polymer degradability^30^. Optical density measurements indicated that degradation in bacterial medium was accompanied by a color change from opaque to transparent, demonstrating rapid breakdown to low-molecular-weight products within 24 h (Supplementary Figs. 6 and 7). Similar degradation kinetics were observed for P3 and P4 polymers (Supplementary Figs. 8 and 9). Hydrolysis-driven degradation (abiotic factor) was faster than bacterial growth (biotic factor), obscuring the relative biodegradability of the polymer (Supplementary Fig. 10).

The physiochemical properties of PAE polymers and the MPs are also adaptable for the intended applications. We designed a group of PAE polymers with glass transition temperatures higher than room temperature (25 °C), which is important to maintain MP structures in household storage conditions. Notably, the solubility of the P5 polymer was low in neutral aqueous solution and dramatically increased under acidic conditions (Fig. 2e). This feature allowed particle formation and structural maintenance in ambient aqueous condition. The response of solubility to pH decrease also enables release of encapsulated cargo in the human stomach for food-related applications. In addition, the morphology of P5 MPs changed notably throughout the water-boiling process as identified by SEM imaging characterization (Fig. 2f–i). Starting with the spherical structure, the MPs fused with each other into amorphous materials during extensive boiling in water. The breakdown of MP structure indicated a macroscopic level of degradation. Chemically, the polymer underwent hydrolysis, breaking down into smaller molecules during the water boiling treatment. Physically, the temperature of the boiling water exceeded the glass transition temperature of the P5 polymer. These two factors collectively led to the structural degradation of P5 MPs. Overall, these results further validate the degradability of the PAE material and their potential as an environmentally friendly replacement of nondegradable microplastic materials.

## Alternative to microbeads with P5 MPs in cleansing products

We explored using degradable P5 MPs to replace microplastics as exfoliating microbeads in personal care products^13,17^. Microplastics in cosmetics are intentionally added and account for 2% (30,000 tonnes) of the annual primary microplastics released into the ocean^17^. Owing to regulatory restrictions, finding environmentally friendly alternatives is urgent^31^. Microbeads in cosmetics require suitable mechanical strength, water resistance, a smooth surface, uniform size and effective cleansing efficiency while minimizing adsorption of persistent organic pollutants^12,20^. We hypothesized that P5 MPs could meet these requirements.

**Fig. 3:**
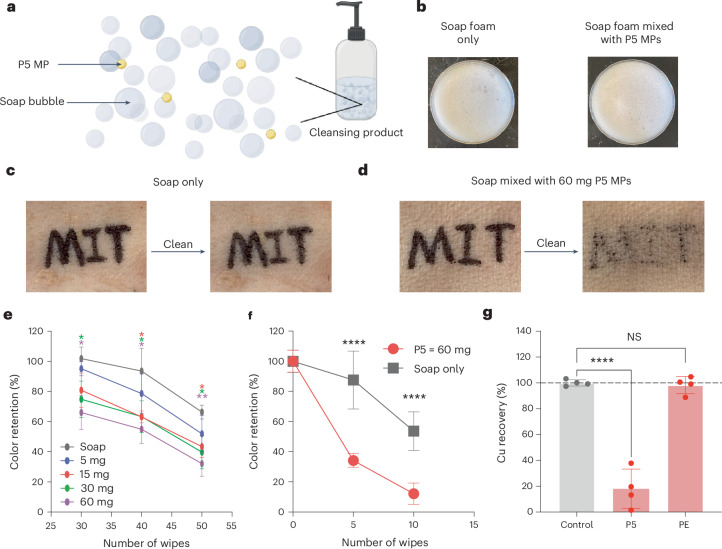
Use of P5 MPs as an alternative to microbeads in cleansing products. **a**, A schematic of a representative cleansing product in this study. P5 MPs were well mixed with soap foam, and then the mixture was applied on skin samples to mimic the cleaning actions. **b**, The addition of a milligram level of P5 MPs did not change the physical appearance of the soap foam (5 ml). **c,d**, Application (50 times) of soap foam mixed with of 60 mg P5 MPs (**c**) was notably effective to clean Sharpie permanent marker patterns on the pig skin samples in contrast to that of soap foam only (**d**). **e**, The color retention decreased with an increase in the concentration of P5 MPs and number of wiping actions applied on the skin samples (*n* = 3 independent replicates; 30 wipes: 30 mg versus soap, *P* = 0.0311, 60 mg versus soap, *P* = 0.0105; 40 wipes: 15 mg versus soap, *P* = 0.0282, 30 mg versus soap, *P* = 0.0470, 60 mg versus soap, *P* = 0.0199; 50 wipes: 15 mg versus soap, *P* = 0.0101, 30 mg versus soap, *P* = 0.0180, 60 mg versus soap, *P* = 0.0035). **f**, P5 MP-added soap foam also more effectively cleaned patterned from a representative cosmetic product (an eyeliner) on the pig skin samples (*n* = 9 independent replicates; 5 wipes, *P* = 4.7118 × 10^−7^; 10 wipes, *P* = 2.4422 × 10^−7^). **g**, P5 MPs showed significantly more effective absorption of copper compared with the polyethylene (PE) MP control (*n* = 4 independent replicates; control versus P5, *P* = 4.0625 × 10^−5^; control versus PE, *P* = 7.0904 × 10^−5^). The data are presented as mean ± s.d. Statistical significance was evaluated using two-tailed Student’s *t*-test. *P* ≤ 0.05 is statistically significant, with *P ≤ 0.05, **P ≤ 0.01, ***P ≤ 0.001 and ****P ≤ 0.0001. NS, not significant. Source data

We tested the cleansing efficiency of P5 MPs by incorporating them into soap foam and removing Sharpie permanent marker and eyeliner stains from pig skin, a suitable model for human skin^32^. We simulated the use of cosmetic products with P5 MPs for cleansing purposes by adding the P5 MPs into a representative cleanser, soap foam, and then applying the mixture on pig skin samples drawn with patterns by a permanent marker (Fig. 3a). The soap foam was essentially a mass of stabilized air bubbles formed by introducing air into soap water and separated from the soap liquid (Supplementary Fig. 11). In the first experiment using a Sharpie permanent marker, the cleanser was fabricated by soap foam (5 ml) and P5 MPs (5–60 mg). The addition of P5 MPs did not change the physical appearance of the soap foam (Fig. 3b and Supplementary Fig. 12) owing to the minimal size and amount of P5 MPs.

To simulate cleansing behavior, the marker pattern was cleaned with wipes soaked with soap foam, with or without P5 MPs. With soap only, the mark was barely removed after wiping 50 times, while the mark was removed 2.4 times more effectively by soap foam mixed with P5 MPs (Fig. 3c,d). Image analysis software was used to analyze color retention and therefore quantify the cleansing efficiency (Supplementary Fig. 13). The color retention decreased as the number of wipes and the mass of P5 MPs increased (Fig. 3e). At the highest P5 MP mass we experimented, 60 mg of P5 MPs in soap foam presented 25.8% color retention after 50 times of the wipe actions, a significant removal rate compared with 61.8% color retention in the soap-only condition. The abrasion effect of P5 MPs could be attributed to both physical and chemical means. Physically, the spherical and regular shape of P5 MPs (Fig. 1i,j) contributed to balanced exfoliation compared with irregularly shaped microbeads used in current cosmetic products^12^. Chemically, based on previous studies, it is likely that the amine group in P5 polymer exhibits high affinity for organic compounds, some of which are present in the Sharpie ink^12^.

Next, we evaluated the cleaning efficiency of P5 MP-added soap for an application-relevant scenario: removal of a cosmetic eyeliner. The same cleaning procedures were followed as the experiment using a Sharpie marker, but a fewer number of wipes were needed to achieve cleaning efficacy. Specifically, wiping five and ten times with P5 MP-added soap foam led to 34% and 12% of color retention, respectively (Fig. 3f and Supplementary Fig. 14). Similarly to the Sharpie experiment, these retention rates were significantly lower than cleaning with only soap, which gave 88% and 54% for wiping five and ten times, respectively.

We extended the cleaning experiment to compare P5 MP-added soap with other materials. Biodegradable polyesters, such as poly(butylene succinate) (PBS) and polycaprolactone (PCL), are widely studied as sustainable alternatives to nondegradable microbeads^33^. We selected PBS, PCL and PCL diol for MP fabrication using the same emulsion-based method, evaluating their morphology via SEM imaging (Supplementary Fig. 15). Under these conditions, the polyesters formed amorphous or spherical MPs with rough surfaces, inferior to the well-defined smooth morphology of P5 MPs. Among the polyesters, PCL had the best morphology and was selected for further cleaning experiments. We also tested polyethylene powder, a representative microplastic, and a commercial exfoliant with carnauba wax. The cleaning actions on these patterns were performed and analyzed in the same manner as previously described for P5 MPs and soap only. All products showed efficiency similar to soap alone, except for P5 MPs, which demonstrated better cleaning (Supplementary Figs. 14, 16 and 17). This enhanced performance is probably due to the improved morphology of P5 MPs, as materials such as PCL, PBS and waxes may suffer from inadequate mechanical strength and functionality^34^.

Another consideration for cleansing efficacy is how effectively cleaning products absorb potentially toxic elements (PTEs), including trace elements and heavy metals, which exist extensively in fine dust and could pose health risks^12,35^. Here, we selected copper as a model PTE. After incubation in aqueous solution of copper(II) sulfate, only 18.0% of copper was recovered from the supernatant, giving 82% absorption, while the samples of polyethylene MPs left with 98.3% of copper, showing essentially no absorption (Fig. 3g). The absorption capacity of copper by P5 MPs was calculated to be 135.8 mg per gram of PAE MPs, considerably higher than chitosan-based microbeads reported in literature^12^. The absorption was also observed visually by color change of the MP samples (Supplementary Fig. 18). Such superior performance in terms of heavy metal absorption could be attributed to chelation between the metal cations and the amine group in P5 polymer^36^. In addition, phenanthrene was used as a model of persistent organic pollutants in seawater^20^. This adsorption study showed that P5 MPs absorbed 28% of phenanthrene in solution and performed noninferiorly compared with the polyethylene MPs (Supplementary Fig. 19).

## Micronutrient stabilization and oral delivery with P5 MPs

The second application of degradable P5 MPs is for stabilizing and delivering micronutrients for food fortification. Micronutrient deficiencies affect about 2 billion people globally, with food fortification being an effective mitigation approach^37,38^. A major challenge is the sensitivity of key micronutrients, such as vitamin A (VA), to heat and light, common in cooking and storage^39,40^. The lack of cold-chain transport in marginalized communities further emphasizes the need for stabilization under long-term storage. Efficient dissociation of micronutrients in the digestive system is also required for bioavailability. Microencapsulation remains a promising method for these needs^41,42^.

Previously, MPs made from poly(butylmethacrylate-*co*-(2-dimethylaminoethyl)methacrylate-*co*-methylmethacrylate) (1:2:1) (BMC) were effective but nondegradable, limiting practical application^14,43^. We hypothesized that the degradable, natural product-based PAE MPs could potentially mitigate environmental risks while stabilizing micronutrients. VA was selected as a model nutrient owing to its vulnerability to high temperatures and social importance, affecting approximately 30% of children under 5 years old, leading to blindness and immune dysfunction^44^.

**Fig. 4:**
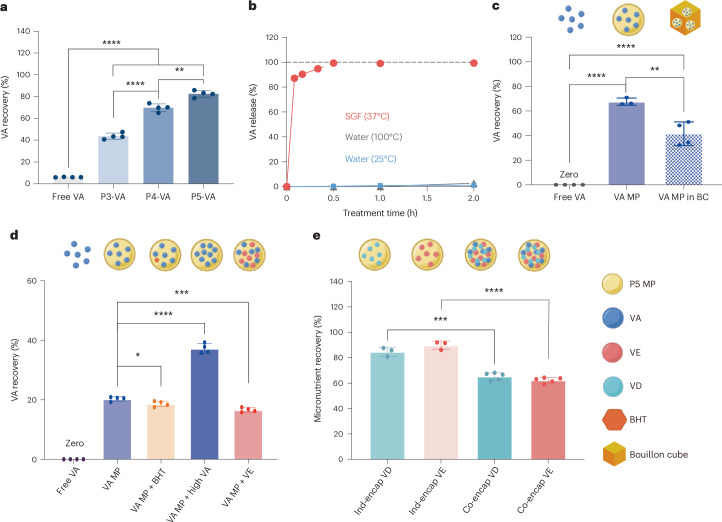
Stabilization and controlled release of micronutrients by P5 MPs. **a**, P3, P4 and P5 MPs effectively stabilized VA after 2 h boiling in water compared with free-form VA, with P5 MPs showing the highest VA recovery (*n* = 4 independent replicates). **b**, Controlled release of VA from P5 MPs when treated with room temperature water, boiling water and 37 °C SGF (*n* = 6 independent replicates). **c**, P5 MPs provided over 60% protection to VA after 6 months storage under 25 °C and 40% relative humidity (RH) condition, a significant improvement compared with free-form VA. The VA recovery was over 40% when the P5 MPs were added in bouillon cubes. VA MP, default P5-VA MP; VA MP in BC, default P5-VA MP in bouillon cubes (BCs) (*n* = 4 for free VA and VA MP in BC; *n* = 3 for VA MP; all replicates were independent). **d**, Under a harsher storage condition at 40 °C and 75% RH, various P5 MP formulations also protected VA in the long term. VA MP + BHT: P5-VA MPs with 0.5% BHT (w/w); VA MP + high VA: P5-VA MPs with VA:P5 1:7 w/w; VA MP + vitamin E (VE): P5-VA MPs with VE, 1:1 VA:VE w/w and 1:5 cargo:P5 w/w (*n* = 4 independent replicates). **e**, P5 MPs stabilized two other hydrophobic micronutrients, vitamins D (VD) and E (VE), in boiling water in individual and collective encapsulated form (*n* = 3 for Ind-encap VD and Ind-encap VE; *n* = 5 for Co-encap VD and Co-encap; all replicates were independent). The data are presented as mean ± s.d. Source data

Improving the nutritional value of fortified food requires the PAE MPs to encapsulate, stabilize and release the micronutrients at the desired conditions. To evaluate the effects of MPs on VA stabilization, we fabricated P3-, P4- and P5-based MPs encapsulated with VA (VA:PAE 1:10 w/w), boiled the product in water for 2 h as a representative cooking practice and measured the VA recovery. Notably, all three compositions significantly enhanced the stability of VA by 7- to 14-fold, giving 44–83% recovery with encapsulation compared with only 6% recovery of the free-form VA (Fig. 4a). Among the three selected polymers, P3 demonstrated a higher VA loading capacity compared with the other two (Supplementary Table 1). However, this higher loading did not compensate for its lower protection efficiency, resulting in the P5 polymer outperforming its counterparts in terms of the total quantity of stabilized VA. A time-course experiment further demonstrated the protection efficiency of VA encapsulated by P5 MPs (Supplementary Fig. 20). Improved VA stabilization correlated with increased TDP feed ratio, with P5 MPs showing the highest VA recovery at 83%. This enhancement can be attributed to elevated hydrophobic interactions between the P5 polymer and VA. In addition, the increasing structural integrity of P3, P4 and P5 MPs also contributed to the highest protection observed in P5 MPs (Supplementary Fig. 21). P5 MPs with VA had a slightly larger diameter (101.6 ± 17.0 µm) compared with those without VA (Supplementary Fig. 22). Importantly, the intrinsic degradability of P5 polymer was not affected by encapsulation, with 9.3% remaining after 2 h boiling (Supplementary Fig. 23). Furthermore, P5 MPs provided strong protection against oxidation, making antioxidant additives such as butylated hydroxytoluene (BHT) unnecessary to achieve high VA recovery (Supplementary Fig. 24). We evaluated VA loading effects on protection efficiency. P5 MPs showed >88% VA recovery for low-to-medium loading and >63% for high loading after 2 h boiling (Supplementary Fig. 25). Under light exposure, P5 MPs provided 60% VA recovery compared with 41% for free-form VA demonstrating effective photoprotection (Supplementary Fig. 26). Overall, the high loading capacity and protection against photolysis of VA rendered P5 MPs remarkably efficient and suitable for micronutrient-fortified food products in practical applications.

To evaluate the release of VA from P5 MPs, we tested three conditions: room temperature water (25 °C), boiling water (100 °C) and 37 °C simulated gastric fluid (SGF). A desired controlled release profile for oral delivery requires the cargo to only be released under the SGF condition. After incubating P5-VA MP samples under each condition for up to 2 h, no notable VA release was observed under room temperature or boiling water conditions. Such minimal release of VA from MPs, even after 2 h treatment in boiling water, is surprising in a positive way, given that P5 MPs without cargo degraded chemically and structurally under the same condition. To explain this observation, we conducted computational simulations, which revealed strong molecular interaction forces between VA and P5, even at simulated high temperatures with short chains of P5 molecules. More details are included in the section ‘MD simulations for P5 polymer applications’. In stark contrast, VA was rapidly released in 37 °C SGF, where 90% of encapsulated VA was detected after 10 min (Fig. 4b). This result is consistent with the pH-dependent solubility change of PAE in aqueous solutions (Fig. 2e). Collectively, these results indicate that, upon oral consumption, the pH sensitivity of the P5 MPs allows a fast dissociation between the P5 polymer and VA molecules, showing promising potential for high bioavailability in humans^14,43^.

We compared VA stabilization and release using other materials, including BMC and biodegradable natural polymers such as chitosan and lignin^14,45,46^. P5 MPs showed higher or comparable VA recovery after 2 h boiling compared with BMC (Supplementary Fig. 27). Chitosan exhibited almost no VA release in SGF, while lignin showed 9–27% release and 45–62% recovery after boiling (Supplementary Table 2). Incorporating soy protein into lignin improved release to 57%, but recovery remained low (34%) compared with P5 MPs (83%). Overall, P5 MPs demonstrated improved VA release and recovery in SGF, showing promise for food fortification.

Another key challenge in global implementation of food fortification is the long-term storage of MP-stabilized micronutrients independent of cold-chain system and vacuum packaging. To assess the long-term stabilization of VA by MPs, the samples were directly exposed to air under various controlled temperatures and humidity. Under the condition of room temperature and 40% relative humidity, P5 MPs rendered 68% VA recovery after 6 months storage, a significant improvement compared with no detectable recovery from free-form VA (Fig. 4c and Supplementary Fig. 28). Notably, free-form VA was completely degraded even after only 1 month storage under this condition, while 88% of VA was recovered from P5 MPs at the same time point (Supplementary Fig. 28). To further mimic real-life scenarios of cooking after storage, we treated 6-month-stored P5-VA samples with 2 h boiling in water, affording 48% VA recovery (Supplementary Fig. 28). We further assessed stability of VA encapsulated in P5-VA MPs within real food matrices, specifically bouillon cubes, which are commonly used in areas with high micronutrient deficiencies^47^. By incorporating P5-VA MPs into bouillon cubes, we mimicked commercial products to test the performance of the MPs in complex conditions (Supplementary Fig. 29). The P5 MPs effectively protected VA during storage in bouillon cubes, maintaining 42% of VA after 6 months (Fig. 4c and Supplementary Fig. 30). Even after boiling the cubes for 2 h, the VA recovery remained consistent, demonstrating robust stabilization capability of P5 MPs to VA (Supplementary Fig. 30).

We expanded the formulations to include (1) BHT, (2) higher VA loading and (3) double fortification with vitamin E. We also tested the protection efficiency of P5 MPs for VA under harsher storage at 40 °C and 75% humidity. Under these conditions, P5 MPs with higher VA loading (VA:P5 1:7) outperformed other formulations, achieving 37% recovery after 6 months (Fig. 4d and Supplementary Fig. 31). After 2 h boiling, 27% recovery was maintained (Supplementary Fig. 31). Higher VA recovery at room temperature and 40% relative humidity compared with 40 °C and 75% humidity was probably due to the P5 glass transition temperature (40 °C), maintaining MP shape under room temperature (Supplementary Fig. 32). The decrease in VA protection after boiling was probably due to P5 degradation. Overall, P5 MPs provided enhanced VA protection in long-term storage, compared with less than 1 month shelf life for free-form VA. Notably, previous studies have shown that traditional high-dose VA supplementation failed to be a long-term solution to treat VA deficiency, which could be attributed to the upper limit in what the body is able to process when receiving a high dose of VA in a short time^48–51^. Evidence has shown that a 6-month high-dose VA supplementation could increase serum retinol only in a limited and transient manner^48,49,51^. A long-term frequent intake of a physiological dose of VA, however, is a more effective measure to stably increase and maintain serum retinol and effectively reduce VA deficiency^48,49,51^. Therefore, fortifying local food products with P5 MPs could be effective in long-term VA stabilization and dosage under common household storage and cooking conditions.

We also evaluated the capacity of P5 MPs to encapsulate, stabilize and release other key micronutrients, including vitamins D and E, both oil-soluble like VA. Using the same fabrication method, we successfully encapsulated vitamins D and E in P5 MPs (Supplementary Table 1). After 2 h boiling, vitamin D recovery was 85% with P5 MPs, compared with 3% for free-form vitamin D (Fig. 4e and Supplementary Figs. 33 and 34). In SGF at 37 °C, 99% of vitamin D was released after 30 min (Supplementary Fig. 35). For vitamin E, recovery improved from 80% to 90%, with 85% release in SGF (Fig. 4e and Supplementary Figs. 33, 36 and 37). Vitamins A, D and E were also successfully co-encapsulated, showing similar stability and release profiles (Fig. 4e and Supplementary Figs. 38 and 39).

We also encapsulated vitamin C palmitate (VCP), achieving 47% recovery after boiling, compared with no recovery for the free form, with 91% release in SGF (Supplementary Figs. 40 and 41 and Supplementary Table 1). We further expanded the encapsulated micronutrients to include iron (as ferrous sulfate) and zinc (as zinc sulfate). Owing to the insolubility of metal salts in organic solvents, we used a reverse emulsion method to create precursor MPs containing the salts with polyvinyl alcohol as an excipient^14^. These precursor MPs were then encapsulated in P5 MPs (Supplementary Table 1). SEM imaging confirmed their morphology (Supplementary Fig. 42). P5 MPs showed 42% recovery and 84% release for iron, while zinc showed nearly 100% recovery and release (Supplementary Figs. 43–46).

## Synthesis scalability and safety evaluation of P5 polymers

We assessed the feasibility and reproducibility of larger-scale synthesis and fabrication, critical for the commercial viability of P5 polymer and its MPs. Polymer quality was evaluated through molecular weight and impurity levels compared with US Food and Drug Administration (FDA) standards, including monomers, byproducts, solvents and reagents (Supplementary Table 3). Estimated daily intake of P5 polymer for VA fortification was determined to be 0.011 g (ref. ^14^). Impurity levels were calculated following FDA standards for food products based on the estimated daily intake (Supplementary Table 3).

Two synthesis rounds were performed: three parallel 10 g batches and a 100 g batch. Molecular weight was consistent across all batches, with impurity levels below permissible thresholds (Supplementary Tables 4 and 5). Large-scale fabrication of MPs using emulsion or spray-drying methods has been well established, including successful BMC MP production for clinical trials^14,52^. These results support the scalability and feasibility of P5 polymer for commercial MP production.

The safety profile of P5 polymer was assessed, focusing on dosages relevant to food fortification. Cytotoxicity studies were conducted using the Caco-2 cell line^53^. Based on the EDI, the relevant dosage range was 0–200 µg ml^−1^ (refs. ^54,55^). No cytotoxicity (cell viability >80%) was observed for raw or degraded P5 polymer (Supplementary Fig. 47). Similar results were obtained for raw or boiled P5-VA MPs at the same concentration range (Supplementary Fig. 48). When VA was tested in free form, encapsulated in P5 MPs or boiled after encapsulation, all formulations exhibited minimal toxicity (Supplementary Fig. 49). Interestingly, VA cytotoxicity decreased when formulated with P5 polymer, suggesting a protective effect. Compared with the BMC polymer, a generally recognized as safe (GRAS) status material, P5 polymer demonstrated a better safety profile (Supplementary Fig. 50). To further evaluate safety, we tested higher concentrations (up to 10 mg ml^−1^) using Caco-2 and HEK-293 cell lines^53,56^. No cytotoxicity was observed for raw P5 polymer up to 1.25 mg ml^−1^, maintaining >80% cell viability (Supplementary Fig. 51). The degradation byproducts of P5 showed no toxicity, with >80% cell viability up to 2.5 mg ml^−1^ in Caco-2 cells and 5 mg ml^−1^ in HEK-293 cells (Supplementary Fig. 52). These results further support the safety of P5 polymer for food fortification applications.

## MD simulations for P5 polymer applications

**Fig. 5:**
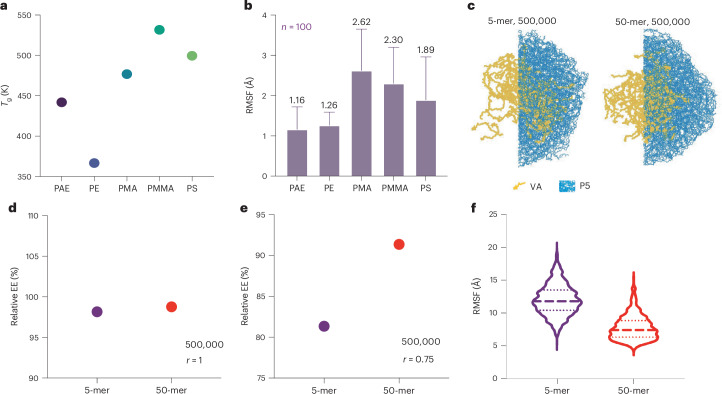
MD simulations for mechanistic study of P5-VA MPs. **a**,**b**, *T*_g_ simulation (**a**) and RMSF results (**b**) of P5 polymer and comparison with common nondegradable microplastic polymers (*n* = 100 independent replicates; data are presented as mean ± s.d.). PE, polyethylene; PMA, poly(methyl acrylate); PMMA, poly(methyl methacrylate); PS, polystyrene. **c**, Snapshots of P5 polymer globule with VA core under 500 K for 5-mer (left) and 50-mer (right). **d**,**e**, The relative encapsulation efficiency (EE) of VA at 500 K for inner sphere with *r* = 1 (**d**) and *r* = 0.75 (**e**). **f**, RMSF measurement of 5-mer and 50-mer P5 polymer globule with VA core. Source data

Molecular dynamics (MD) simulations were used to study the properties of P5 polymer for the two identified applications, comparing its thermodynamics with nonbiodegradable polymers commonly used as microplastics, such as poly(methyl methacrylate), polyethylene, poly(methyl acrylate) and polystyrene^13^. We calculated the glass transition temperature (*T*_g_) and root-mean-squared fluctuation (RMSF) of P5 polymer to understand its thermal and structural stability^57,58^. *T*_g_ is critical for determining mechanical and thermal properties, while RMSF measures polymer solidity, similar to its application in assessing protein stability^59^. The P5 polymer showed a low *T*_g_ and comparable RMSF to other plastics, suggesting practical processability and mechanical properties (Fig. 5a,b).

To understand VA stabilization with P5 polymer, we analyzed the interaction between the polymer and VA molecules. Two types of behavior were observed: VA either adsorbed on the polymer surface or was surrounded by polymer chains within the globule (Supplementary Fig. 53). The small surface area-to-volume ratio of the MPs resulted in most VA being encapsulated, as supported by fluorescence imaging showing homogeneous VA distribution across P5-VA MPs (Supplementary Fig. 54). We constructed a core–shell structure of P5 polymer globule with VA molecules in the center (‘core’) as the initial structure for the simulation (Supplementary Fig. 55). These initial structures enabled us to study VA encapsulation and distribution within the MP, excluding molecules potentially located on the surface after MP fabrication. To capture the continuous spectrum of polymer degradation, we selected three chain lengths (50-mer, 20-mer and 5-mer) as representative of various degradation states and two temperatures (300 K = room temperature, and 500 K = in silico temperature of boiling water (*T*_b_, _water_)). Each simulation ran for 1 μs to trace the evolution of the encapsulation morphology. We maintained a consistent size of the polymer globule across different chain lengths by adjusting the number of P5 polymers along with 150 VA molecules (Supplementary Table 6). The encapsulation effect was quantified using the relative encapsulation efficiency, defined as the percentage of heavy atoms of VA molecules residing within a relative cutoff distance (*r* < *r*_cutoff_) to the globule center of mass (COM) over all VA heavy atoms in the distance (Supplementary Fig. 56). The distance was normalized by the globule radius, as the surface of globule was defined as *R* = 1. At both room temperature and *T*_b, water_, the relative encapsulation efficiencies of VA were over 98% at *R* = 1 across all chain lengths, indicating that VA molecules were well encapsulated within the MP and that the water interaction was shielded from VA molecules (Fig. 5c,d and Supplementary Figs. 57 and 58). This observation indicated that a high molecular weight was not necessary for VA molecules encapsulation at room temperature and even *T*_b, water_.

Since almost all VA molecules were ‘in’ the MP with minimum water interaction, we investigated why the degraded oligomer (for example, 5-mer) had lower but still substantial recovery of VA molecules compared with the nondegraded polymers (for example, 50-mer). We hypothesized that VA molecules consistently move radially outward, and the shorter chain at a higher temperature has a larger diffusivity to transport the VA molecules close to the surface more easily; moreover, VA molecules are more susceptible to be degraded by water. Since the initial structure of the core–shell MP with VA molecules has a relative encapsulation efficiency of 1 at *r* < 0.5, the relative encapsulation efficiency at a certain distance cutoff (for example, *r* < 0.75) after 1 μs simulation was a measure of the diffusivity of the VA molecules in the polymer. A lower encapsulation efficiency indicates a larger diffusivity as more VA molecules could be found outside the cutoff (*R* = 0.75 < *r* < *R* = 1). At room temperature, the relative encapsulation efficiency of different chain lengths was 100% and indifferentiable as the majority of the monomers were immobile (RMSF <10 A) (Supplementary Figs. 57 and 58), that is, the chain length did not substantially affect VA diffusion within the polymer globule at room temperature. At *T*_b, water_, the relative encapsulation efficiency of 5-mer was lower than that of 20-mer and 50-mer, suggesting that VA molecules were more diffusive in the 5-mer case (Fig. 5c,e and Supplementary Figs. 57 and 58). The promoted mobility of VA molecules originated from a larger fraction of the mobile monomers for 5-mer at *T*_b, water_ (Fig. 5f). Meanwhile, even in this condition, the fraction of immobile monomers was around 23% that hindered the movement of VA molecules. Experimentally, we observed that the P5 polymer demonstrated protection efficacy for VA in boiling water, even when the polymer was in a highly degraded state. Specifically, we induced degradation of the P5 polymer by boiling it in water, then formulated the degraded polymer with VA using the same MP fabrication method. As expected, no MPs were formed, probably due to the insufficient hydrophobicity of the two degradation byproducts to support MP formation, yet amorphous solid material was still obtained after freeze-drying, as confirmed by visual inspection and SEM imaging (Supplementary Fig. 59). Notably, after a 2 h boiling treatment, VA recovery rates were 55.7% and 37.1% when using P5 degradation byproducts and isosorbide alone in the formulation, respectively (Supplementary Fig. 60). These results suggest that the addition of P5 polymer, even in a highly degraded form, aids, to some extent, in the protection of VA stability, compared with the free-form VA, which showed only a 6% recovery. These findings align with the observations from the MD simulation work.

The simulation was expanded to include P1 polymer, comparing its RMSF with that of P5. The results showed higher RMSF for P1, indicating greater molecular mobility and lower hydrophobicity, which is consistent with experimental observations of P1 failing to form MPs while P5 successfully did (Supplementary Figs. 61 and 62). The lower hydrophobicity of P1, stemming from its monomer composition, supports the conclusion that the properties of P5 are more suited for MP formation and VA encapsulation. Collectively, these findings provide a mechanistic foundation for understanding the ability of P5 MPs to protect VA and offer insights for further optimizing PAE MPs.

## Discussion

Microplastic pollution is an exigent environmental and health issue that is raising global concerns. The European Union and multiple countries, including the USA and the UK, have implemented strict regulations on products that heavily and intentionally use microplastic materials, including a ban on the use of microplastics in personal care rinse-off products and micronutrient encapsulation for food fortification^11,12^. Since limited success has been achieved by end-of-the-pipe strategies such as filtration during wastewater treatment, replacing nondegradable microplastics with environmental-friendly degradable materials offers a potentially more sustainable and effective approach to mitigate microplastic pollution. In this study, we presented the development of a potentially sustainable approach to combat global microplastic pollution: a natural product-inspired degradable PAE MP platform with potential to replace microplastics in cosmetic products and for oral micronutrient delivery. The PAE polymers presented high degradability and satisfactory material properties, including hydrophobicity and pH sensitivity, to meet demands of the two distinct applications. The potential scalability of established polymer synthesis and MP fabrication further places this category of polymers as potential materials for global use as an alternative of nondegradable microplastic materials.

## Methods

### PAE synthesis

The PAE polymers were synthesized as previously described^22^. For the synthesis of P5 polymer, isosorbide diacrylate (1.00 g, 3.93 mmol) and TDP (0.82 g, 3.93 mmol) were dissolved in tetrahydrofuran (THF, 5 ml) with BHT (1 mg ml^−1^) and stirred at 50 °C for 48 h to synthesize poly[(isosorbide diacrylate)-*co*-TDP] (P5). The mixture was then cooled to room temperature and diluted to 0.05 mg ml^−1^ with THF. To end-cap, TDP (1 g, 4.75 mmol) was dissolved in THF (1 ml) and then added to the mixture, reacting for an additional hour. The product was subsequently precipitated into hexane and dried under vacuum. Other PAE polymer compositions were synthesized following the same procedures with different monomer feed ratios of piperazine to TDP. In the scalability studies, hexane was replaced by heptane for better synthesis performance.

### PAE MP fabrication and characterization

PAE MPs were prepared by a modified oil–water emulsion method, as described previously^14^. For the fabrication of P5 MPs with 10%wt VA loading, P5 polymer (200 mg) and 10 mg VA were dissolved in DCM (2 ml). The resulting organic phase was then emulsified in 40 ml of polyvinyl alcohol solution (1%) with a stirring rate of 500 rpm for 2 h, followed by decanting into 150 ml deionized water stirring at 500 rpm for 10 min. MPs were collected by gravity and then washed with deionized water. The final MP product was obtained via lyophilization. The successful formation of solid MPs was determined depending on whether solid material could be collected from aqueous phase after dispersion of the organic phase. In the cases of P5 MPs individually encapsulated with 10%wt of vitamins A, D, E or VCP, the above procedure was used; in the cases of P5 MPs with BHT, BHT was also dissolved in the organic phase; in the case of P5 MPs encapsulated with different VA loading, the mass of VA in the organic phase was changed accordingly; in the case of P5 MPs collectively encapsulated with vitamins A, D and E, 10 mg of each micronutrient was dissolved in the organic phase; in the case of P5 MPs encapsulated iron or zinc, 10 mg of the corresponding precursor MPs was dissolved in the organic phase; other steps were the same as described above. Fabrication methods of precursor MPs are detailed in Supplementary Information. Polyester MPs were fabricated following the same method as PAE MPs.

### Water contact angle measurement of PAE polymer

P3, P4 and P5 polymers (200 mg) were dissolved in DCM (1 ml) and solvent-casted on a glass slide covered with vinyl Bytac Teflon. Polymer films were formed after DCM evaporated. A droplet of water (2.5 µl) was dispensed on the polymer film with controlled dispensing height. The water droplet images were captured by a Drop Shape Analyzer (Kruss DSA100). The circle fitting method was used to analyze the water contact angle.

### Characterization of P5 polymer degradation in boiling water

P5 MP samples (5 mg) were weighed in an Eppendorf tube and suspended in 1 ml of water. The sample tubes were placed on a thermomixer (Eppendorf ThermoMixer C) for treatment at 100 °C with stirring (500 rpm) for different time periods, followed by drying via lyophilization. For NMR, polymer content in the boiled product was dissolved in deuterated chloroform. BHT was added as an internal standard. Peak integration of PAE polymer (5.20 ppm) and BHT (5.01 ppm) was used to calculate the percentage of polymer content left in the boiled product for characterization of PAE degradation. For GPC analysis, the polymer content in the boiled product was dissolved in THF and characterized via light scattering characterization. Degradation of P5 polymer in P5 MPs with 10%wt VA loading was characterized by the same method.

### Determination of cleansing efficiency by P5 MPs in rinse-off products

To prepare the representative soap-based cleansing product, a commercially available soap base was added into water (2.5 mg ml^−1^). Vigorous vortex or foam dispenser used in foaming hand soap products was used to introduce air into soap water. The foam was then separated from liquid and collected for later use. Next, P5 MPs were added to and well mixed with the soap foams. To mimic stains on human skins in real-life scenarios, patterns were drawn on cadaver pig skin samples using a Sharpie permanent marker or a representative eyeliner. Kimwipes were fully soaked with soap foam products and then used to wipe the targeted areas on the skin samples to mimic cleansing actions. The patterns on skin samples were recorded in form of pictures before and after cleansing, with the number of wiping actions noted. A reference mark was used to minimize variance between pictures. ImageJ was used to analyze color retention rate of the pattern on skin samples before and after the cleansing process. The pictures were set to 16-bit followed by profile plotting.

### Characterization of PTE absorption

Copper, in the form of copper(II) sulfate, was selected as a model PTE^12^. Copper(II) sulfate aqueous solution (2.5 mg ml^−1^) was prepared, and then 5 mg of P5 or polyethylene MPs were added to 1 ml of the solution. The samples were incubated on a thermomixer (Eppendorf ThermoMixer C) under 25 °C and 1,000 rpm for 24 h, followed by centrifugation at 12,000*g* for 5 min. The copper concentration in the supernatant was analyzed by inductively coupled plasma optical emission spectrometry (ICP-OES; Agilent ICP-OES 5100 VDV). The absorption capacity was calculated using

$$A=\frac{\left({C}_{0}-{C}_{{\mathrm{e}}}\right)V}{m},$$

where $A$ is the absorption capacity; ${C}_{0}$ and ${C}_{{\mathrm{e}}}$ are the initial and equilibrium copper ion concentration, respectively; $m$ is the mass of MPs; and $V$ is the solution volume.

### Quantification of micronutrients

Vitamins A, D and E were quantified by high-performance liquid chromatography (HPLC; Agilent Infinity 1260 II HPLC) using a C-18 column (AcclaimTM PolarAdvantage II, 3 μm, 4.6 × 150 mm) with detection by a diode array detector at 325 nm, 265 nm and 290 nm, respectively. Isocratic elution of a mobile phase of acetonitrile with a flow rate of 0.5 ml min^−1^ was used. Vitamin C was analyzed using BioVision colorimetric assay kit. Iron and zinc were characterized via ICP-OES (Agilent ICP-OES 5100 VDV).

For the cases of vitamins A, D and E, the incubated MP products were dissolved in 0.9 ml THF with 0.1% BHT and 0.1 ml water, followed by mixing and centrifugation (27,000*g*, 5 min). HPLC samples were prepared by adding 0.1 ml supernatant to 0.9 ml acetonitrile with 0.1% BHT. For the cases of vitamin C, the incubated MP products were dissolved in 1 ml SGF completely and then characterized by colorimetric assay kit. The free-form vitamins A, D, E and VCP samples were processed following the same procedures for stability characterization. For the cases of iron and zinc, the incubated MP products were completely dissolved in 1% HCl and then quantified by ICP-OES.

### Treatment of PAE MPs with micronutrients in boiling water, room temperature water and SGF

To evaluate micronutrient stability in boiling water, room temperature water or SGF, 5 mg of P5 MPs of different formulations were measured into microcentrifuge tubes and dispersed in 1 ml of either water or SGF. The samples were placed on a thermomixer (Eppendorf ThermoMixer C) for stirring (500 rpm) and incubating (100 °C for boiling water, 25 °C for room temperature water and 37 °C for SGF) for different time periods. Subsequently, the water-incubated MP product was dried via lyophilization for next-step characterization. The SGF-incubated product was first centrifuged at 27,000*g* for 10 min at each time point, followed by removal of supernatant, and the pellet was lyophilized. The obtained samples were then prepared for HPLC, colorimetric assay kit or ICP-OES characterization.

### Exposure of P5 MPs with VA under simulated sunlight

To evaluate the stability of VA under simulated sunlight, a 300 W Xenon arc-based solar simulator was used (Solar Light, model 16S-300-002). The irradiance at the focus point was measured to be 150 mW cm^−2^ using a radiometer (Solar Light, model PMA2100). Open-cap, flat-bottom scintillation vials with P5 MPs with 10%wt VA at the vial bottom were placed at the focus point of the simulated sunlight, where the vials were cooled with an ice bath during light exposure. The MP samples were irradiated for 10 min or 20 min before HPLC characterization.

### Long-term storage of P5 MPs

P5 MPs with 10%wt VA were placed in microcentrifuge tubes and stored at 40 °C and 75% humidity. The temperature and humidity were controlled in a storage chamber connected with a condensate recirculation system (Caron, Environmental Chamber and Condensate Recirculator). At predetermined time points, the samples were removed from the storage chamber and prepared for HPLC characterization. Other formulations including (1) with 10%wt VA and 0.5%wt BHT, (2) 15%wt VA, (3) with 10%wt VA and 10%wt vitamin E and (4) P5 MPs with 10%wt VA stored under 25 °C and 40% humidity were treated following the same procedures.

### Cytotoxicity analysis of P5 polymer, BMC and VA

Caco-2 cells (ATCC) were cultured in Eagle’s Minimum Essential Medium (ATCC) supplemented with fetal bovine serum (Invitrogen) to a final concentration of 20% and antibiotics (Invitrogen) to a final concentration of 1%. HEK-293 cells (ATCC) were cultured in Dulbecco’s modified Eagle medium (DMEM; Invitrogen) with 10% fetal bovine serum and 1% antibiotic. A total of 10,000 cells were seeded in each well of a 96-well plate in 100 µl of full growth medium. After 24 h of incubation, the medium was replaced with 100 µl of fresh medium containing the test articles, either raw or degraded P5 polymer, raw or boiled P5-VA MPs, BMC or free-form VA, at various concentrations. The cells were then incubated for an additional 24 h. Cell viability was assessed using the CellTiter-Fluor Cell Viability Assay kit (Promega).

### MD simulation of P5 polymer with VA

The protocols for simulating multichain polymer melts and single-chain aqueous solutions were adapted from prior work^57,58^. The general Amber force field was applied, and all molecules and monomers were assigned partial charges from restrained electrostatic potential charges using Gaussian 16 Revision C.01^60,61^. AmberTools19 was employed to parameterize the polymers and assemble monomers into extended chains to be simulated using Amber18. The temperature was controlled in all simulations using a Langevin thermostat with a collision frequency of 2 ps^−1^ for all simulations^62^. Bond lengths were constrained using the SHAKE algorithm with bonds involving hydrogen^63^. When pressure was specified, an isotropic Berendsen barostat was used with a time constant 1 ps (ref. ^64^). The simulation time step was set to 2 fs with a MD leap-frog integrator. Electrostatic interactions were calculated using particle mesh Ewald method with a real-space cutoff of 8.0 Å (ref. ^65^). The van der Waals interaction cutoff was also set to 8.0 Å. Periodic boundary conditions were used in all directions.

Three single-chain PAEs with a degree of polymerization 5, 20 or 50 (5-mer, 20-mer or 50-mer) were collapsed in water with the generalized Born/surface area implicit solvent model after an annealing cycle (650 K to 300 K in 20 ns) to relax and collapse the structure^66^. Sixty vitamin A palmitate (VAP) molecules were placed in a sphere with a radius of 30 Å at the center of simulation box, then 150/38/15 PAE 5-mer/20-mer/50-mer were placed in a sphere with a radius or 100 Å using PACKMOL, resulting in a loose packed initial structure^67^. The system was heated at 650 K for 20 ns in vacuum, leading to a core–shell PAE–VAP nanoparticle. VAPs are constrained in the center to prevent the mixing of VAP and PAEs. The PAE–VAP nanoparticle was then solvated in extended simple point charge (SPC/E) water. All the simulations began with a short minimization, initial heating and equilibration under constant pressure. The 1 μs production runs at 300 K, 350 K, 400 K, 450 K and 500 K were under 1 bar.

The encapsulation efficiency was defined as the number of VAP heavy atoms (carbon and oxygen atoms) inside a certain distance cutoff from the COM of the nanoparticle with a normalization factor of all VAP heavy atoms. The distance cutoff was normalized with the radius of gyration of the PAE–VAP nanoparticle so that a relative distance equal to 0 indicates the COM and 1 indicates the surface of the nanoparticle. For any system, the encapsulation efficiency is 0 at the relative distance equal to 0 and is 1 at the relative distance equal to 1. The relative encapsulation efficiency is the encapsulation efficiency normalized at 300 K of the same nanoparticle system.

RMSF was calculated for each heavy atom in PAE monomer in the 100 conformations. Translational and rotational motion was first removed using the root-mean-square deviation for all PAE atoms with the mean structure as the reference.

All analysis was performed using a combination of AmberTools19 and Python package pytraj (a package binding to Amber’s *cpptraj* program). The last 100 ns trajectory was used for analysis^68,69^.

### Statistical analysis

All quantitative measurements were performed with at least three independent replicates. The data are presented as mean ± s.d. Statistical significance was evaluated using two-tailed Student’s *t*-test. *P* ≤ 0.05 is statistically significant, with *P ≤ 0.05, **P ≤ 0.01, ***P ≤ 0.001 and ****P ≤ 0.0001.

### Reporting summary

Further information on research design is available in the Nature Portfolio Reporting Summary linked to this article.

## Supplementary information

Supplementary Information Supplementary Materials and Methods, Supplementary Figs. 1–62, Supplementary Tables 1–6, NMR spectra and GPC curves.

Reporting Summary

## Source data

Source Data Fig. 1 Statistical source data.

Source Data Fig. 2 Statistical source data.

Source Data Fig. 3 Statistical source data.

Source Data Fig. 4 Statistical source data.

Source Data Fig. 5 Statistical source data.

## Data Availability

Data supporting the findings of this study are available within the Article and its Supplementary Information. Source data are provided with this paper and via Figshare at 10.6084/m9.figshare.27216447 (ref. ^70^).
