# Supplementary material for: Degradable poly(β-amino ester) microparticles for cleansing products and food fortification
Source: Nat Chem Eng. 2024 Dec 6;2(1):77–89. doi: 10.1038/s44286-024-00151-0 (PMC11782087; doi:10.1038/s44286-024-00151-0)
Supplement: Supplementary file 1 — Supplementary Materials and Methods, Supplementary Figs. 1–62, Supplementary Tables 1–6, NMR spectra and GPC curves. [file 44286_2024_151_MOESM1_ESM.pdf]

nature chemical engineering

Supplementary information https://doi.org/10.1038/s44286-024-00151-0

# Degradable poly(β-amino ester) microparticles for cleansing products and food fortification

In the format provided by the authors and unedited

**Table of content**


Materials and Methods …………………………………………………………………………2

Supplementary Figures ……………………………………………………………………...9

Supplementary Tables ……………………………………………………………………...71

NMR Spectra …………………………………………………………………………………78

GPC Curves ………………………………………………………………………………...84

## Materials and Methods

**Materials.** All materials were purchased from Sigma unless specified otherwise. The Vitamin A (retinyl palmitate) was from Alfa Aesar. SGF was purchased from Ricca Chemical Company. The soap base was purchased from Amazon with ingredients of Glycerin, Water, Sodium Stearate, Propylene Glycol, Sorbitol, Sodium Laurate, Sodium Laureth Sulfate, Sodium Chloride, Stearic Acid, Lauric Acid, Titanium Dioxide, Sodium Thiosulfate, Pentasodium Pentetate, and Tetrasodium Etidronate. The commercial exfoliant (Burt's Bees Gentle Facial Scrub for Sensitive Skin with Aloe Vera) and commercial eyeliner (e.l.f. Expert Liquid Liner) were purchased from Amazon.

**Synthesis of isosorbide diacrylate (2,5-di-O-acryloyl-1,4:3,6-dianhydro-D-glucitol).** A round bottom flask was charged with isosorbide (10 g, 68.4 mmol) and 4-dimethylaminopyridine (0.836 g, 6.8 mmol) dissolved in 200 mL of dichloromethane (DCM), followed by the addition of triethylamine (17.310 g, 171 mmol). The reaction mixture was cooled to 0 °C with stirring. Acryloyl chloride (15.48 g, 171 mmol) was dissolved in 60 mL DCM and then added to the reaction mixture via a dropping funnel. The reaction mixture was stirred for 24 hours after adding acryloyl chloride with temperature rising to room temperature gradually. The reaction mixture was washed with 2 times of 1M HCl and brine, followed by drying of the organic phase with sodium sulfate. The crude product was then purified by flash column chromatography to afford the pure product as white solid (90% yield). $^{1}$H NMR (400 MHz, dimethyl sulfoxide-d6) δ (ppm): 6.38-6.34 (dt, $J$ = 17.3, 1.5 Hz, 2H), 6.25-6.16 (m, 2H), 6.00 (dd, $J$ = 3.7, 1.4 Hz, 1H), 5.98 (dd, $J$ = 3.7 Hz, 1.4 Hz, 1H), 5.19 (dd, $J$ = 9.8, 5.4 Hz, 1H), 5.14 (d, $J$ = 3.2 Hz, 1H), 4.83 (t, $J$ = 5.3 Hz, 1H), 4.46 (d, $J$ = 5.0 Hz, 1H), 3.89,-3.80 (m, 4H). $^{13}$C NMR (126 MHz, dimethyl sulfoxide-d6) δ 164.74, 164.68, 132.46, 132.10, 127.88, 127.83, 85.46, 80.50, 77.75, 73.91, 72.39, 70.23, 39.86, 39.69, 39.52, 39.35, 39.19.

**Characterizations of PAE polymer.** The absolute molecular weight (MW) characterization was performed on an Agilent PL-220 high temperature gel permeation chromatograph (GPC) equipped with a heated autosampler, thermostatted column oven, a retractive index (RI) detector and a light scattering detector. The column used for the separation was an Agilent PLgel Mixed-C column. The mobile phase was tetrahydrofuran (THF), and the run was carried out at flow rate of 1 mL/min.

Sample solutions were prepared at 10 mg/mL. The number-averaged MW (Mn), weight-averaged MW (Mw), and polydispersity index (Mw/Mn, PDI) of the P5 polymer were characterized to be 55.0 kDa, 57.4 kDa, and 1.04, respectively. Conventional GPC characterizations were also used to evaluate MW of P5 polymers, including samples treated by boiling water. To accommodate solubility of degradation byproducts, the mobile phase was set as 50:50 (v/v) THF:dimethyl sulfoxide (DMSO), and poly(methyl methacrylate) (PMMA) was used as standards. The Mn, Mw, and PDI of a representative batch of P5 were characterized to be 35.2 kDa, 63.1 kDa, and 1.79, respectively. The same method was used to evaluate MW of P1, P2, P3, and P4 polymers. The Mn, Mw, and PDI of a representative batch of P1 were characterized to be 22.8 kDa, 35.4 kDa, and 1.56, respectively. The Mn, Mw, and PDI of a representative batch of P2 were characterized to be 26.9 kDa, 45.0 kDa, and 1.67, respectively. The Mn, Mw, and PDI of a representative batch of P3 were characterized to be 19.1 kDa, 32.0 kDa, and 1.68, respectively. The Mn, Mw, and PDI of a representative batch of P4 were characterized to be 26.3 kDa, 49.8 kDa, and 1.90, respectively.

$^{1}$H and$^{13}$C NMR spectra were obtained for the P1 through P5 polymer using Bruker Avance Neo spectrometer operating at 500.34 MHz for $^{1}$H($^{13}$C), in $CDCl_3$, and referenced to the residual solvent signal. Spectra for quantitative integration were recorded using 64 scans.

P1 polymer: $^{1}$H NMR (500 MHz, $CDCl_3$) δ 7.24 (s, 2H), 7.16 (d, *J* = 8.3 Hz, 2H), 5.21 (d, *J* = 2.8 Hz, 1H), 5.16 (q, *J* = 5.5 Hz, 1H), 4.81 (t, *J* = 5.1 Hz, 1H), 4.47 (d, *J* = 4.6 Hz, 1H), 3.94 (dt, *J* = 15.8, 4.7 Hz, 3H), 3.80 (dd, *J* = 10.0, 5.2 Hz, 1H), 2.67 (ddt, *J* = 20.8, 7.3, 3.8 Hz, 4H), 2.56 (t, *J* = 7.4 Hz, 2H), 2.49 (dd, *J* = 15.5, 8.3 Hz, 10H), 1.67 (s, 4H). $^{13}$C NMR (126 MHz, $CDCl_3$) δ 171.82, 171.51, 148.97, 148.30, 127.94, 120.34, 85.92, 80.78, 77.98, 77.29, 77.24, 77.03, 76.78, 73.94, 73.42, 70.42, 53.38, 53.35, 52.84, 42.58, 32.31, 32.29, 31.99, 31.98, 30.94.

P2 polymer: $^{1}$H NMR (500 MHz, $CDCl_3$) δ 7.24 (s, 4H), 7.17 (d, *J* = 8.4 Hz, 6H), 5.21 (d, *J* = 2.6 Hz, 1H), 5.16 (q, *J* = 5.6 Hz, 1H), 4.82 (t, *J* = 5.1 Hz, 1H), 4.48 (d, *J* = 4.6 Hz, 1H), 3.95 (dt, *J* = 15.8, 4.8 Hz, 3H), 3.80 (dd, *J* = 9.9, 5.3 Hz, 1H), 2.86 (t, *J* = 10.9 Hz, 1H), 2.72 – 2.65 (m, 2H), 2.57 (q, *J* = 7.2 Hz, 2H), 2.49 (dd, *J* = 14.9, 8.0 Hz, 9H), 1.94 (d, *J* = 10.7 Hz, 1H), 1.68 (s, 10H), 1.27 (m, *J* = 5.4 Hz, 3H), 1.18 (m, *J* = 7.7 Hz, 3H), 0.89 (m, *J* = 6.6 Hz, 2H). $^{13}$C NMR (126 MHz, $CDCl_3$) δ 171.83, 171.51, 152.17, 148.97, 148.30, 127.95, 120.35, 120.32, 85.95, 85.93, 80.78, 77.98, 77.29, 77.24, 77.03, 76.78, 73.95, 73.42, 70.42, 53.82, 53.80, 53.38, 53.35, 52.84, 42.58, 36.75, 35.65, 32.44, 32.40, 32.30, 31.99, 31.61, 30.94, 22.67, 14.14.

P3 polymer: $^{1}$H NMR (500 MHz, $CDCl_3$) δ 5.20 (s, 1H), 5.15 (q, $J$ = 5.6 Hz, 1H), 4.81 (t, $J$ = 5.1 Hz, 1H), 4.47 (d, $J$ = 4.6 Hz, 1H), 4.01 – 3.89 (m, 3H), 3.79 (dd, $J$ = 9.9, 5.3 Hz, 1H), 3.77 – 3.67 (m, 1H), 2.84 (t, $J$ = 10.8 Hz, 2H), 2.74 – 2.61 (m, 4H), 2.59 – 2.42 (m, 5H), 1.93 (q, $J$ = 10.6 Hz, 3H), 1.66 – 1.57 (m, 3H), 1.36 – 1.21 (m, 5H), 1.17 (m, $J$ = 6.7 Hz, 6H), 0.86 (m, $J$ = 10.6, 5.2 Hz, 3H). $^{13}$C NMR (126 MHz, $CDCl_3$) δ 172.09, 171.82, 171.77, 171.51, 85.94, 85.92, 80.77, 78.00, 77.97, 77.92, 77.90, 77.29, 77.24, 77.03, 76.78, 73.94, 73.88, 73.41, 70.42, 70.39, 67.99, 53.82, 53.80, 53.38, 53.35, 52.84, 36.75, 35.65, 32.44, 32.41, 32.30, 32.07, 31.98, 31.60, 25.62, 23.91, 22.67, 22.66, 14.13.

P4 polymer: $^{1}$H NMR (500 MHz, $CDCl_3$) δ 5.20 (d, $J$ = 2.9 Hz, 1H), 5.15 (q, $J$ = 5.6 Hz, 1H), 4.81 (t, $J$ = 5.0 Hz, 1H), 4.47 (d, $J$ = 4.6 Hz, 1H), 4.02 – 3.88 (m, 3H), 3.79 (dd, $J$ = 9.9, 5.2 Hz, 1H), 2.91 – 2.79 (m, 4H), 2.65 (dt, $J$ = 22.7, 7.6 Hz, 4H), 2.57 (t, $J$ = 7.1 Hz, 2H), 2.50 (t, $J$ = 7.1 Hz, 2H), 1.92 (t, $J$ = 10.6 Hz, 3H), 1.69 – 1.57 (m, 4H), 1.27 (m, $J$ = 7.2 Hz, 7H), 1.16 (m, $J$ = 7.7 Hz, 9H), 0.88 (m, $J$ = 6.7 Hz, 4H). $^{13}$C NMR (126 MHz, $CDCl_3$) δ 172.09, 171.83, 171.78, 171.51, 85.94, 80.78, 77.92, 77.29, 77.24, 77.03, 76.78, 73.89, 73.41, 70.42, 53.82, 53.79, 53.38, 53.35, 52.83, 36.75, 35.65, 32.44, 32.41, 32.07, 31.60, 23.91, 22.66, 14.13.

P5 polymer: $^{1}$H NMR (500 MHz, $CDCl_3$) δ 5.19 (d, $J$ = 3.0 Hz, 1H), 5.14 (q, $J$ = 5.5 Hz, 1H), 4.80 (t, $J$ = 5.0 Hz, 1H), 4.46 (d, $J$ = 4.6 Hz, 1H), 3.99 – 3.87 (m, 3H), 3.79 (dd, $J$ = 9.8, 5.2 Hz, 1H), 2.87 – 2.79 (m, 4H), 2.64 (dt, $J$ = 22.9, 7.4 Hz, 5H), 2.56 (t, $J$ = 7.1 Hz, 2H), 2.49 (t, $J$ = 7.2 Hz, 2H), 2.09 – 1.84 (m, 4H), 1.71 – 1.52 (m, 4H), 1.26 (q, $J$ = 4.3 Hz, 3H), 1.13 (s, 1H), 0.86 (q, $J$ = 6.6 Hz, 2H). $^{13}$C NMR (126 MHz, $CDCl_3$) δ 172.09, 171.77, 85.93, 80.78, 77.92, 77.31, 77.05, 76.80, 73.88, 73.41, 70.42, 53.82, 53.79, 36.75, 35.65, 32.45, 32.41, 32.08, 31.59, 23.90, 22.66, 14.14.

Thermogravimetric analysis (TGA) measurements were carried out under nitrogen atmosphere (AirGas, ultra-high purity grade) using a TGA 550 from TA Instruments. The ramp speed was 20 °C min$^{-1}$, and isotherms were performed from room temperature to 500 °C. The degradation temperature ($T_d$) of a polymer is the onset temperature in the TGA curve, where a deflection is first observed from the established baseline prior to the thermal change. Thermal transitions were determined by differential scanning calorimetry (DSC) using a Discovery DSC from TA instruments with powdered samples (5−8 mg) sealed in aluminum pans. Both TGA and DSC analyses were performed for 3 samples and an average was taken for each polymer. The thermal degradation temperature of P3, P4, and P5 polymers was determined to be 262 °C, 263 °C, and

267 °C, respectively. The glass-transition temperature was measured to be 29 °C, 36 °C, and 41 °C, respectively.

The storage modulus (E′) of P5 polymer was determined using the dynamic mechanical analyzer (TA Instruments DMAQ850). Three samples of P5, each with a consistent thickness, were solution-cast. The E′ of each film was measured three times at room temperature and subsequently averaged. The average E′ value was determined to be 390.24 MPa.

P5 polymer was dissolved in THF to prepare three dilute polymer solutions with varying concentrations ($C = 0.5$ wt%, 1 wt% and 2 wt%). The viscosity ($\eta$) of each dilute polymer solution was measured using the AMETEK Brookfield Viscometer (LV Master R204(25C)) at a fixed viscometer speed of 5.0 RPM. Three viscosity measurements were conducted for each dilute solution concentration and the averaged values were obtained. The data obtained from the viscosity measurements were fitted into the truncated Huggins equation which relates the viscosity of the polymer solution to its concentration. The intrinsic viscosity of P5 polymer was determined to be 69.1 dL/g.

The Fourier Transform Infrared (FTIR) spectrum of the P5 polymer was acquired employing a Thermo Fisher IS50R Bench spectrometer. Additionally, Raman spectroscopy was performed on the P5 polymer using a Renishaw Invia Reflex Raman Confocal Microscope, integrated with a Leica Optical Microscope. The excitation source utilized was a 785nm, 100mW Bar Laser. Detection of the Raman signal was facilitated by a 1" CCD array detector, which boasted deep depletion capabilities tailored for the enhanced detection of infrared (IR) wavelengths spanning from 200nm to 1064nm. The resultant spectra were captured at a resolution of 1024 x 256 pixels. The obtained spectra are shown below.

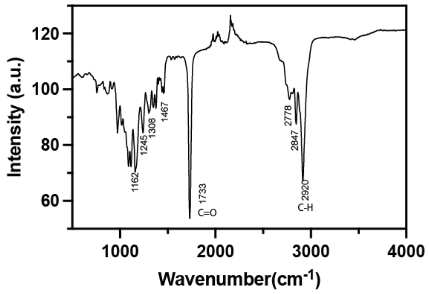


FTIR spectrum of the P5 polymer

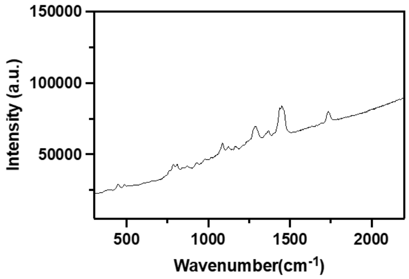


Raman spectrum of the P5 polymer

**Imaging characterization of P5 MP morphology and distribution.** The morphology of MP samples, including (1) P3, P4, and P5 MPs, (2) P3, P4, and P5 MPs with 10% VA loading, (3) P5 MPs after treatment of boiling water and lyophilization, and (4) precursor PVA-Zn MPs and PAE-PVA-Zn MPs, were characterized by SEM (Zeiss Crossbeam 540 SEM). The samples were coated with gold prior to SEM imaging. The size distribution of P5 MPs was evaluated using ImageJ. The SEM images, which contained over 200 counts of MPs, were adjusted to the same scale and B&W, followed by analysis using the Analyze Particles feature. The diameter of individual MPs was then calculated based on particle area. The VA distribution across P5 MPs was characterized by a multiphoton laser scanning confocal microscope (Olympus FV1000). The MPs were dispersed on the surface of a water droplet on a glass slide, and the excitation/emission wavelength was 193/226 nm.

**Biodegradation Analysis.** Biodegradation testing was carried out according to Fransen, Av-Ron, *et al.*[1] Briefly, agar growth media with suspended polymer was prepared by homogenizing 0.8 mL of polymer dissolved in dichloromethane at 100 mg/mL into 15 mL of ATCC growth medium: 179 *Pseudomonas* Medium containing 20 g/L agar using a Scilogexe D500 homogenizer with a with S20F/ER20 probe head. Into 8 of 12 wells in a plate, 0.8 mL of polymer containing ATCC growth medium with agar was added. The remaining 4 wells were filled with 0.8 mL of ATCC growth medium containing no polymer. *Paucimonas lemoignei* was incubated in liquid ATCC growth medium until reaching an OD600 of 0.5-0.8 at 30 °C and 180 rpm. To the center of 6 of the polymer containing wells and 2 of the wells containing no polymer, 1 µL of the *P. lemoignei* liquid culture was added. Plates were incubated for a total of 13 days at 30 °C and monitored via imaging at regular intervals using an Opentron 1st generation robot with a 2.8-12mm Varifocal USB Webcam Mini Camera with adjustable focus and a 8x11 in Kaiser Slimlite Plano light tablet.

Optical density curves showing the progression of degradation in each well were extracted using methods and software developed by Fransen, Av-Ron, *et al*.

**Solubility of P5 MPs in aqueous solutions.** P5 MPs were measured in microcentrifuge tubes, followed by addition of aqueous solutions with different pH. Because P5 polymer was expected to be more soluble in low-pH environment, the following concentrations of P5 MPs in aqueous solutions were used: (1) 40 mg/mL for pH = 1; (2) 20 mg/mL for pH = 3; (3) 10 mg/mL for pH = 5; (4) 7.5 mg/mL for pH = 7. The samples were placed on a thermomixer (Eppendorf ThermoMixer C) for stirring (500 rpm) and stirred at 1000 rpm under 20 °C for 24 hours. Next, the samples were centrifuged at 12k g for 5 minutes to collect undissolved solid, followed by removal of supernatant and lyophilization. The mass of undissolved polymer was measured for solubility calculation.

**Fabrication of bouillon cubes with P5-VA MPs.** The bouillon powder was prepared by grounding of commercially available bouillon cubes (Maggi, Nestle, USA) with a mortar and pestle. P5-MPs (~10 mg) were weighed into a weighing boat and manually mixed with bouillon powder (~1.5 g). Next, a tablet presser was used to press the mixture into a solid cube, reformed into the commercially available form. The ratio of P5-VA MPs to bouillon powder enabled ~0.655 mg VA per gram of bouillon powder.[2] The re-pressed cubes were then wrapped into aluminum foils for long-term storage.

**Evaluation of VA stability in bouillon cubes.** The bouillon cube samples with P5-VA MPs were stored under 25 °C and 40% humidity. At each designated time point, the bouillon cubes were prepared into bouillon soup as a representative practice of real-life cooking. Samples of the storage-only group were added into 20 mL of RT water and stirred for 30 min, which allowed complete breakdown of the bouillon cubes. The other group was added into 20 mL of boiling water and treated for 2 hours. Afterwards, the liquid was freeze fried, and VA in the bouillon cubes was extracted for downstream characterizations. Briefly, the sample was added with 0.5 mL water and 4.5 mL THF, vigorously vortexed, and then centrifuged. Next, the supernatant was diluted into acetonitrile (1:9 w/w) and then characterized by RP-HPLC. The HPLC method was the same as reported for P5-VA MPs.

**Fabrication of pre-MPs encapsulated with iron and zinc.** The pre-MPs were formulated by a modified inverse emulsion process as previously described.[3] Briefly, the water phase was formed by dissolving 100 mg of ferrous sulfate or zinc sulfate with 100 mg of Polyvinyl alcohol (PVA) in 10 mL water. The water phase was then dispersed into an oil phase containing 150 mL mineral oil and 600 μL Span 80. The mixture was first homogenized for 10 minutes using a Silverson L5M-A laboratory mixer (Silverson Machines, Inc.), followed by constant stirring for 24 hours at 45 °C. The obtained pre-MPs were isolated via centrifugation at 1.6k g for 15 minutes, washed by hexane and acetone, and dried under vacuum.

**Characterization of POP absorption.** Phenanthrene (PAT) was used as a model POP chemical.[4] PAT was dissolved in ethanol (2 mg/mL) and then diluted to 5 μg/mL with ethanol/water mixture (2/8, v/v) due to its high hydrophobicity. 5 mg of P5 or PE MPs were added to 1 mL of the PAT solution, followed by incubation on a thermomixer (Eppendorf ThermoMixer C) under 25 °C and 1000 rpm for 24 hours. PAT in the supernatant was then quantified via liquid chromatography-mass spectrometry (LC–MS) (Agilent 6125B MS and Agilent 1260 Infinity LC) using extracted-ion chromatogram. An external standard curve of PAT was constructed for calculation of PAT in the sample supernatant.

**Supplemental Figures**

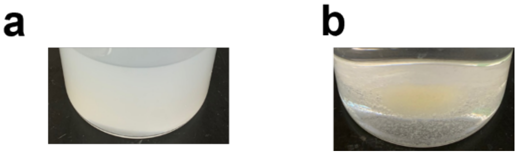

**Supplementary Figure 1. Solid particle formulation from PAE polymers. (a)** After dispersion of an organic phase (a PAE polymer dissolved in dichloromethane solvent), P1 and P2 polymers generated cloudy solutions. **(b)** The same dispersion process with P3, P4, and P5 polymers formed solid particle materials in the water phase.

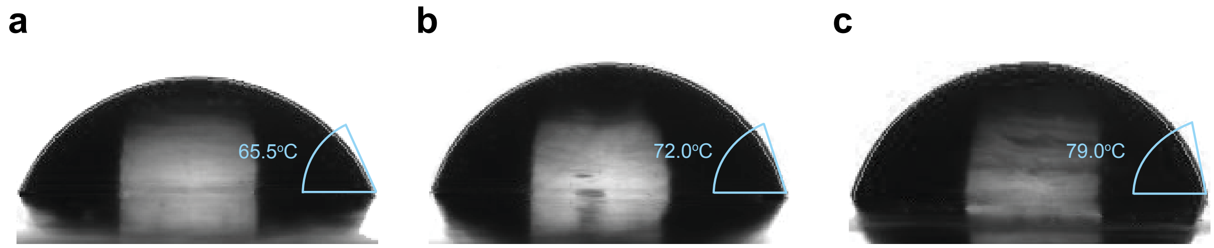


**Supplementary Figure 2. Water contact measurement images for PAE polymers.** Increasing hydrophobicity is demonstrated for P3 (a), P4 (b), and P5 (c) through water contact angle measurements.

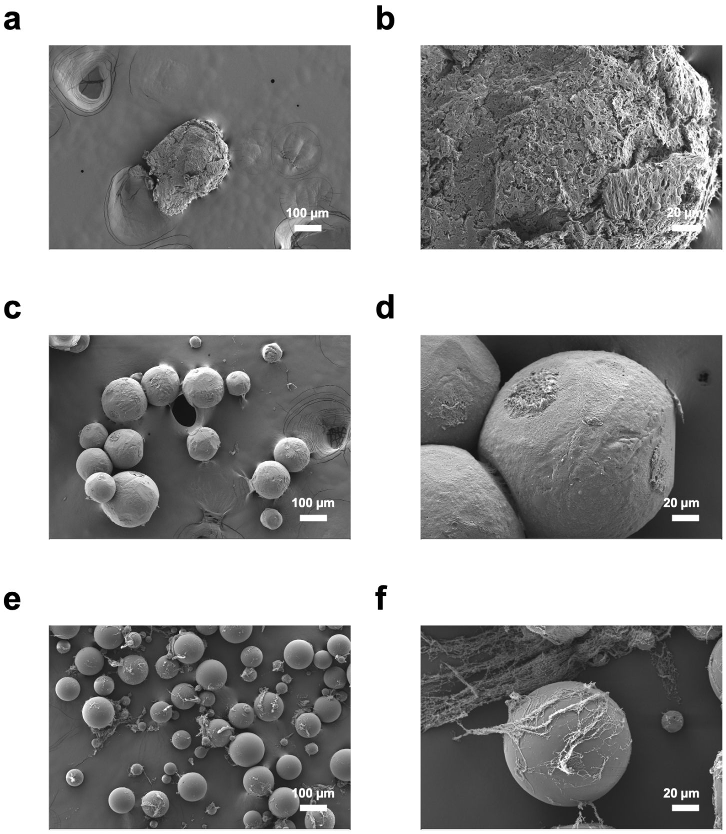


**Supplementary Figure 3. Morphology characterizations of PAE MPs by SEM.** SEM images showing the surface morphology of PAE MPs fabricated using P3 (a, b), P4 (c, d), and P5 (e, f).

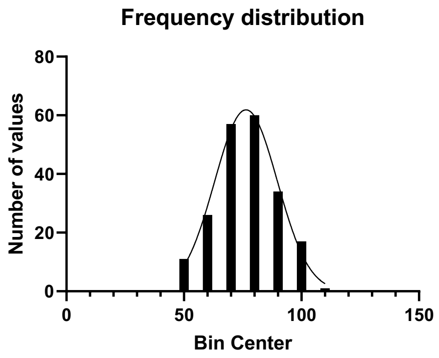


**Supplementary Figure 4. Size distribution of P5 MPs.** Frequency distribution of P5 MP size characterized from SEM images. The value at bin center corresponds to the diameter of MPs in the analyzed SEM images.

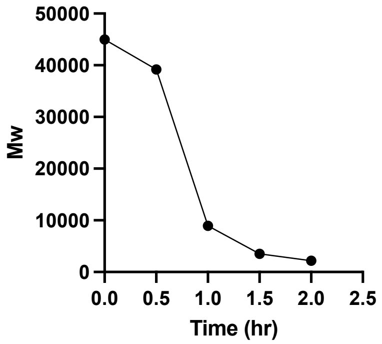


**Supplementary Figure 5. Degradation kinetics of the P5 polymer in boiling water.** Mw results of the P5 polymer throughout the 2-hour boiling test.

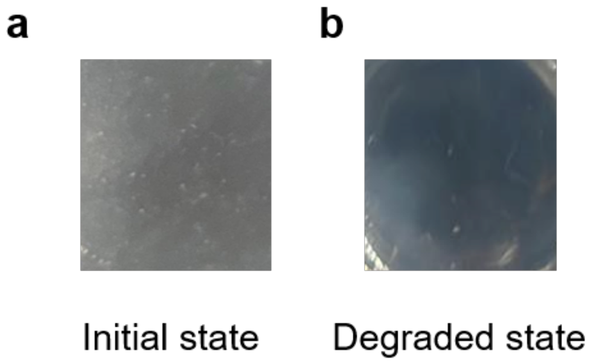


**Supplementary Figure 6. Visual comparison of bacterium medium with initial and degraded polymer material.** Phone pictures of initial state (a) and degraded state (b) of growth medium with P5 polymer. The images visually demonstrated the difference in optical density.

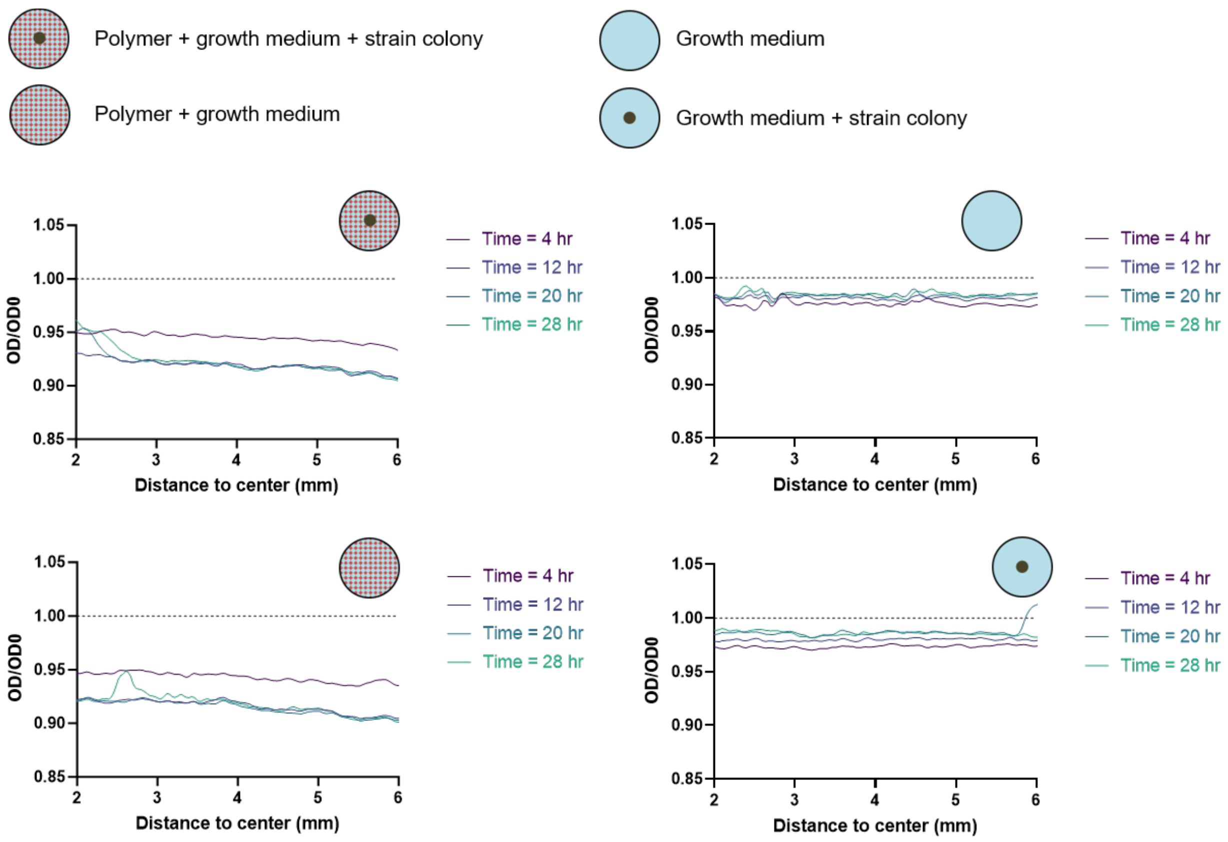


**Supplementary Figure 7. Representative OD curves of the P5 polymer samples in the biodegradation assay.** A total of 4 conditions were run, including (1) polymer added to growth medium inoculated with strain colony, (2) polymer added to growth medium, (3) growth medium only, and (4) growth medium inoculated with strain colony. The values of OD/OD0 below 1 indicated degradation of the P5 polymer in the assay.

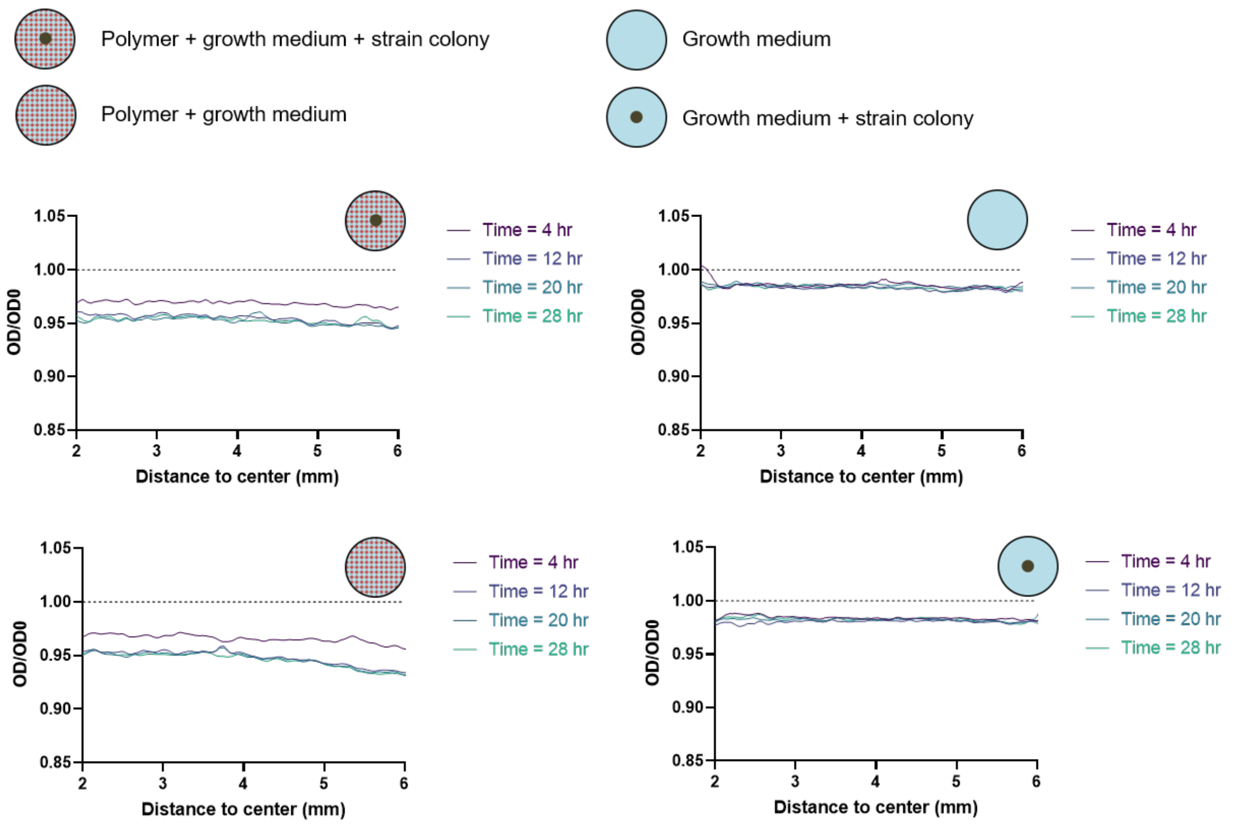


**Supplementary Figure 8. Representative OD curves of the P3 polymer samples in the biodegradation assay.** Light scattering signals dropped to below 1 for the P5 polymer samples in the growth medium with and without strain colonies, which indicates both hydrolysis and biodegradation of the P5 polymer.

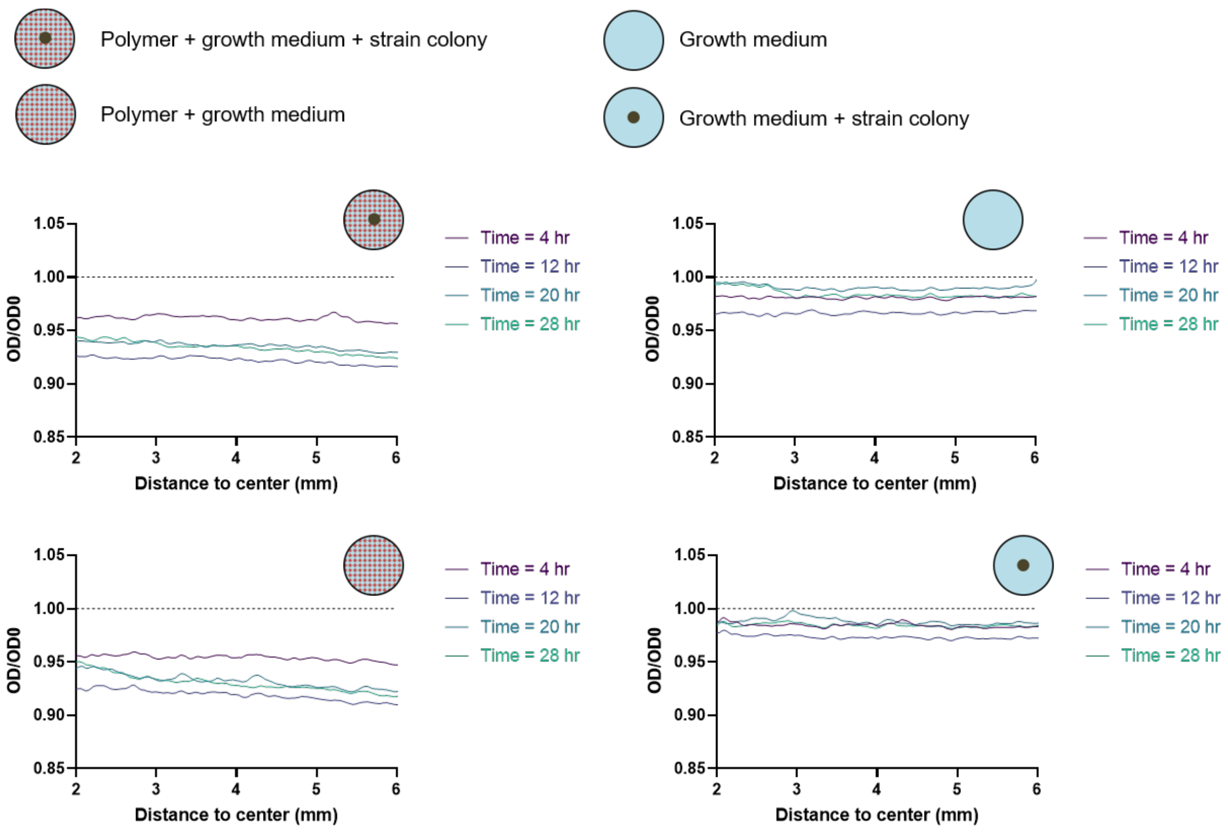


**Supplementary Figure 9. Representative OD curves of the P4 polymer samples in the biodegradation assay.** The values of OD/OD0 dropped to lower than 1, showing degradation of the P4 polymer in the assay.

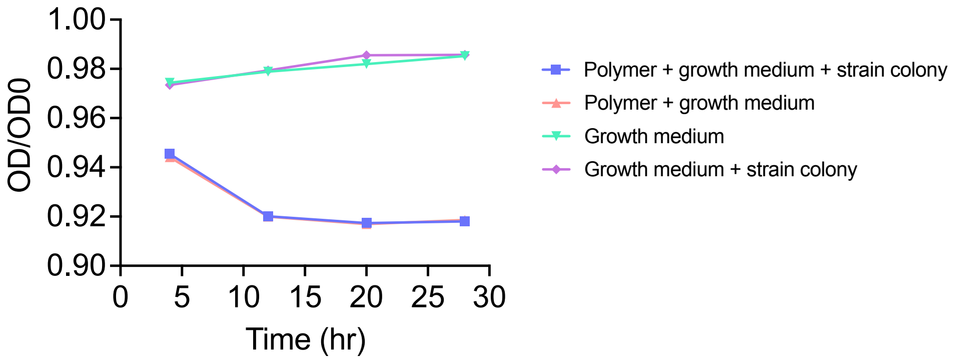


**Supplementary Figure 10. Optical density values of growth medium samples at radius of 4 cm.** The values of optical density for four growth medium samples, including (1) polymer in growth medium with a strain colony, (2) polymer in growth medium, (3) growth medium only, and (4) growth medium with a strain colony, were singled out and plotted for comparison.

a b

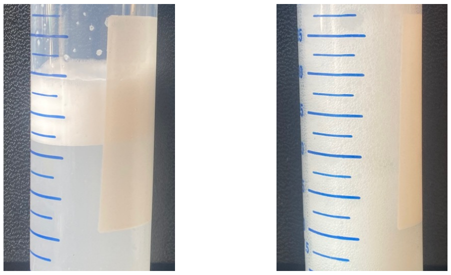

**Supplementary Figure 11. Visual comparison of soap liquid and foam.** (a) Soap foam formed after vigorous vortexing of the soap-water mixture. (b) The soap foam was then separated from the liquid phase and collected for subsequent experiments as a representative cleaning product.

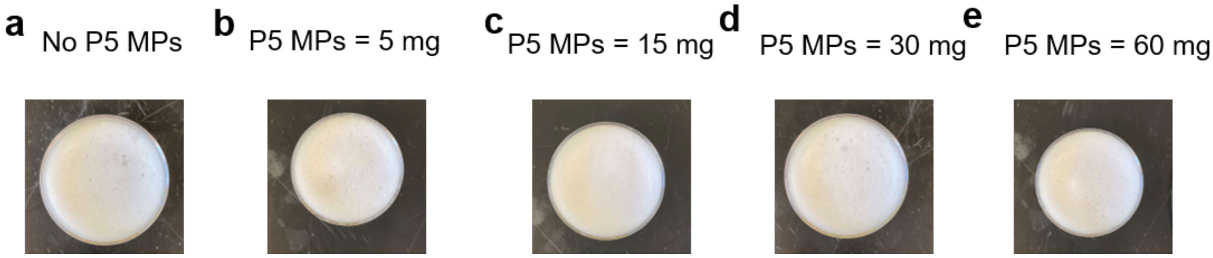


**Supplementary Figure 12. Visual comparison of the representative cleansing products with P5 MPs.** Pictures of soap foam products mixed with **(a)** no P5 MPs, **(b)** 5 mg P5 MPs, **(c)** 15 mg P5 MPs, **(d)** 30 mg P5 MPs, and **(e)** 60 mg P5 MPs. The physical appearance of these products did not change significantly due to small amount of MPs added.

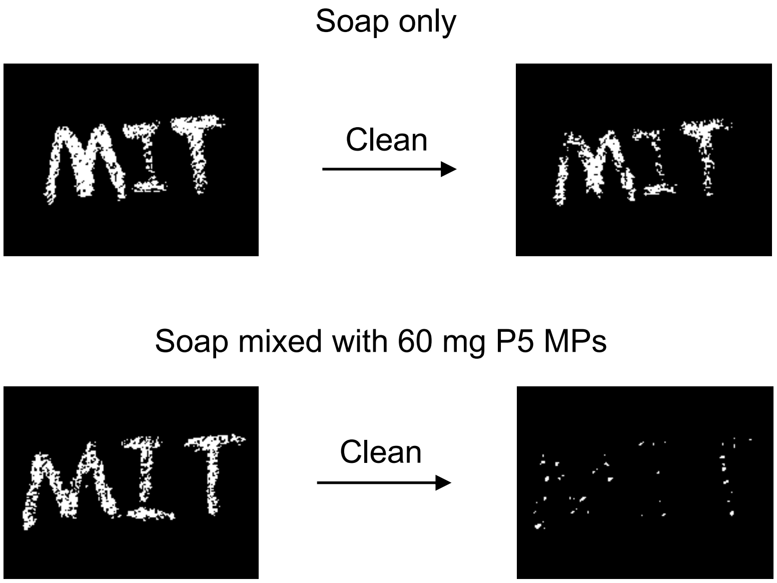


**Supplementary Figure 13. Processed images of Sharpie marks on pig skin samples.** Image analysis software ImageJ was used to process pig skin pictures with marks before and after representative cleaning actions. Cleaning efficacy was quantified by color retention. After 50 times of wiping, soap foam with 60 mg P5 MPs removed significant amount of the Sharpie mark dye compared to the product of only soap foam.

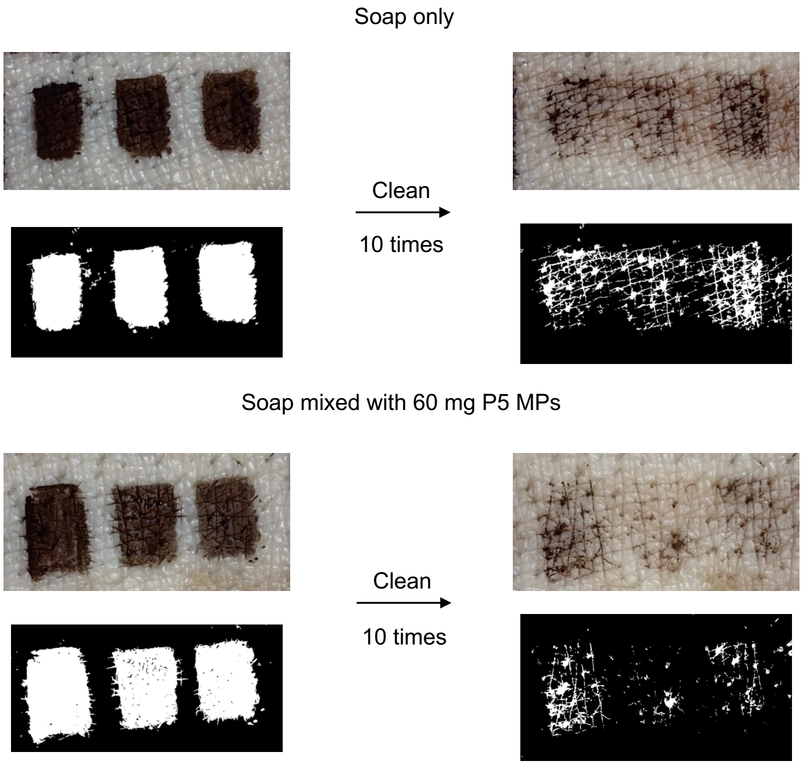


**Supplementary Figure 14. Raw and processed images of eyeliner marks on pig skin samples cleaned by soap foam only or added with P5 MPs.** Pictures of pig skin samples marked by a commercial eyeliner were processed by ImageJ. Color retention was used to quantify cleaning efficacy. After 10 times of wiping, the eyeliner mark was more effectively removed by the soap added with P5 MPs, shown both on photos and software-converted images.

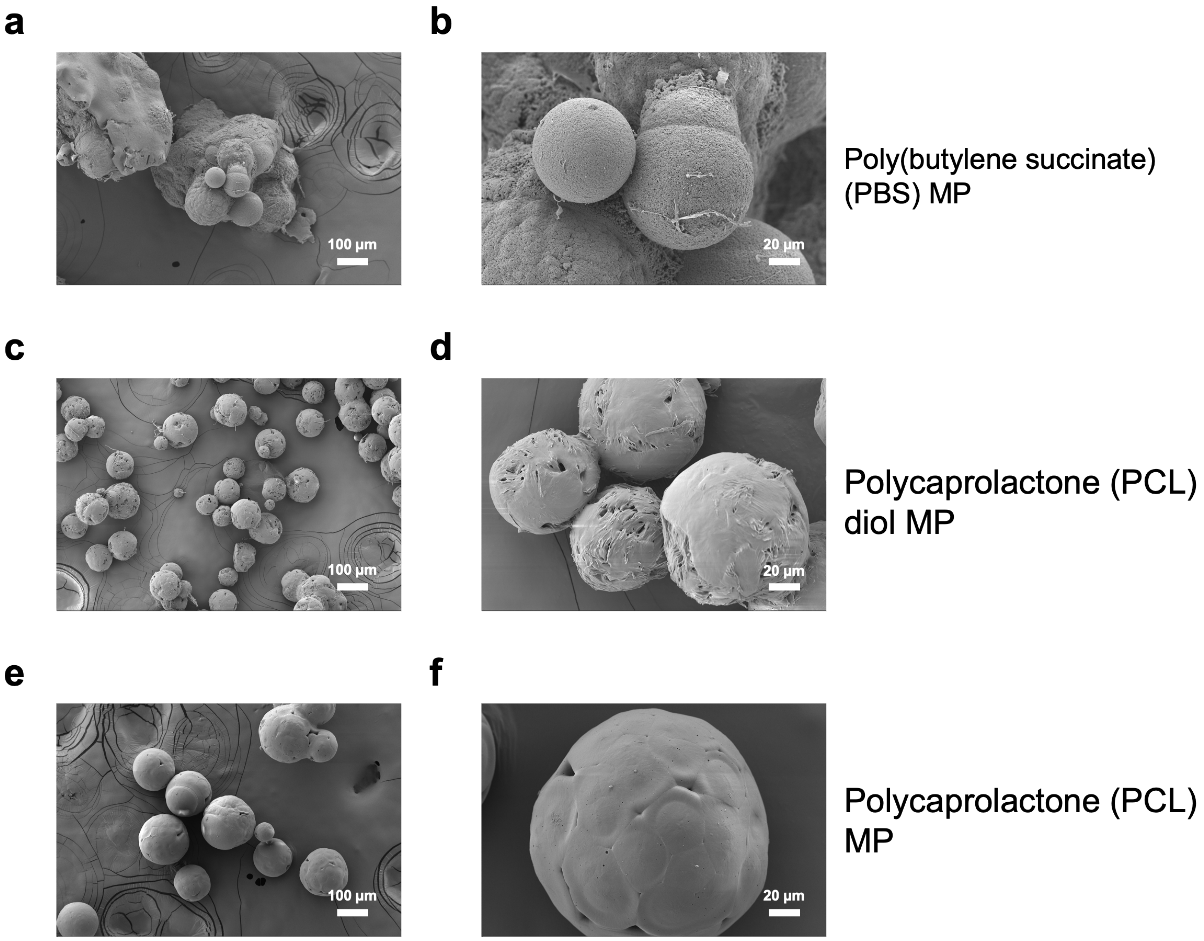


**Supplementary Figure 15. SEM images of MPs made from selected polyesters.** MPs were fabricated using an emulsion-based method with (a) PBS, (b) PCL diol, and (c) PCL. The surface morphology of the obtained MPs was characterized by SEM.

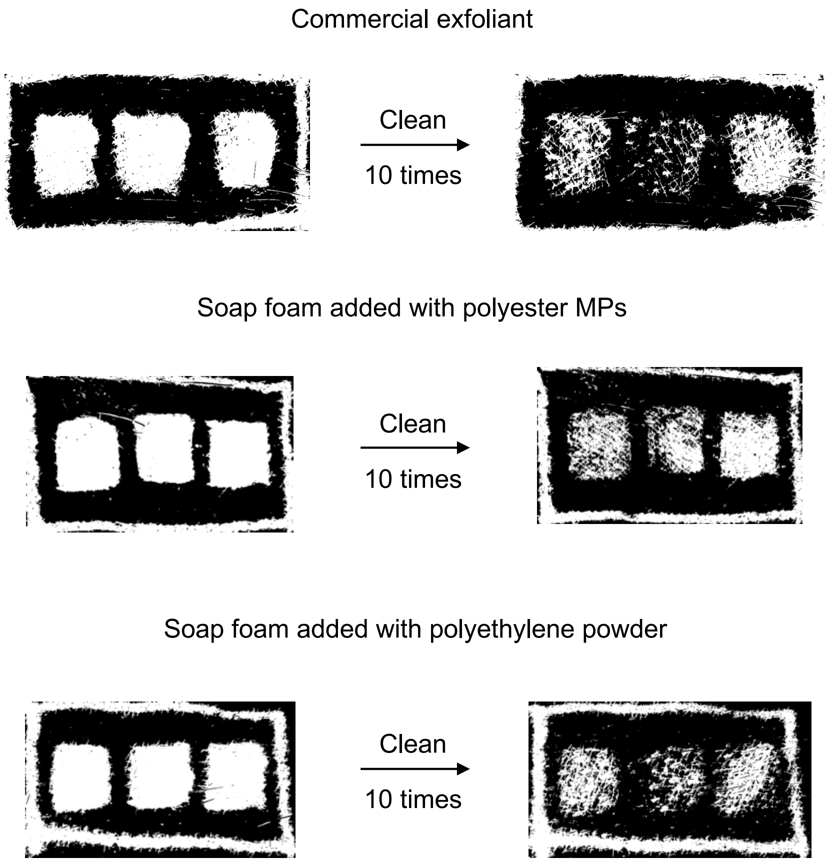


**Supplementary Figure 16. Processed images of eyeliner marks on pig skin samples cleaned by a commercial exfoliant or soap foam containing polyester MPs or polyethylene powder.** Pig skin samples marked with commercial eyeliner were cleaned using a commercial exfoliant, soap foam with polyester MPs (PCL), and soap foam with polyethylene powder. ImageJ was used to quantify color retention before and after the cleaning action.

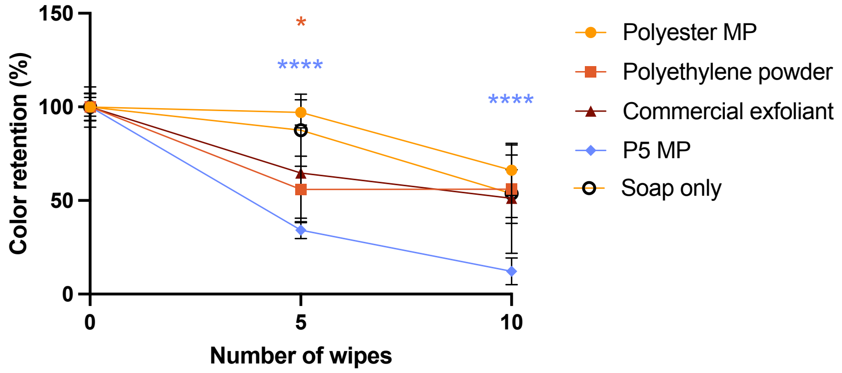


**Supplementary Figure 17. Comparison of cleaning efficiency for eyeliner between P5 MPs and other materials.** Color retention of eyeliner patterns on pig skin was quantified for processed images before and after 5 or 10 wiping actions. The color retention was compared between P5 MPs, soap foam containing polyester MPs or polyethylene powder, a commercial exfoliant, and soap foam alone. n = 3 for Polyester MP, Polyethylene powder, and Commercial exfoliant; n = 9 for P5 MP and Soap only. Data are presented as mean +/- SD. Statistical significance was evaluated using two-tailed Student's *t* test. A $P$ value of $\leq 0.05$ is statistically significant, with * $P \leq 0.05$, ** $P \leq 0.01$, *** $P \leq 0.001$, and **** $P \leq 0.0001$.

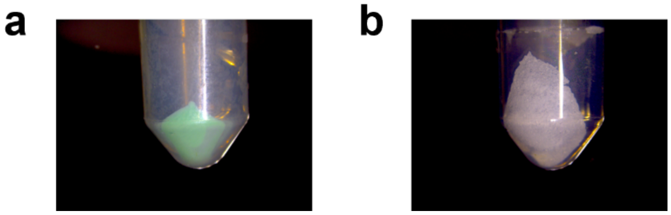

**Supplementary Figure 18. Visual comparison of copper absorption by P5 and PE MPs.** Pictures of (a) P5 MPs and (b) PE MPs after incubation in copper(II) sulfate aqueous solution showed effective absorption of copper by P5 MPs given the color change.

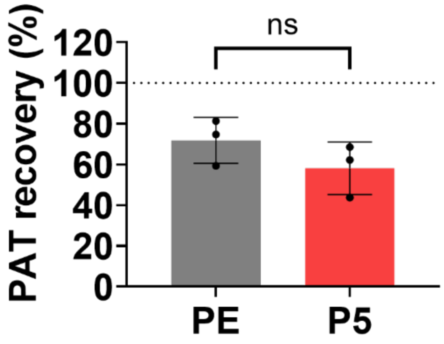


**Supplementary Figure 19. Recovery of PAT from incubation with PE and P5 MPs.** After incubation, P5 MPs showed similar adsorption to PAT compared to the PE MPs as a control. n = 3 independent replicates. Data are presented as mean +/- SD. Statistical significance was evaluated using two-tailed Student's *t* test. A $P$ value of $\leq 0.05$ is statistically significant, with * $P \leq 0.05$, ** $P \leq 0.01$, *** $P \leq 0.001$, and **** $P \leq 0.0001$.

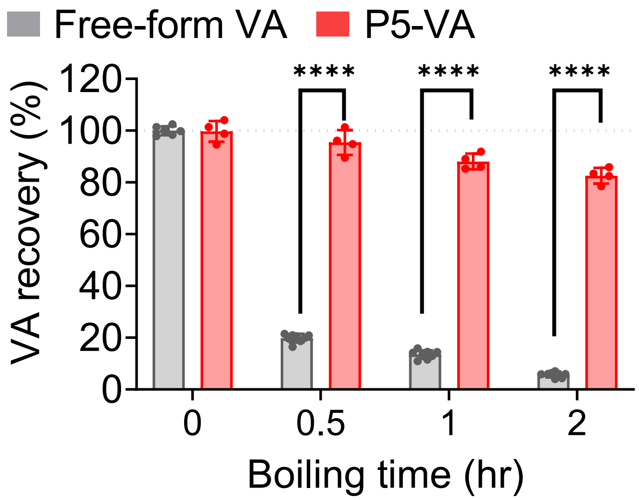


**Supplementary Figure 20. Time course study of P5 MPs stabilizing VA in boiling water.** P5-MPs provided effective protection to VA in boiling water throughout the 2-hour boiling water treatment. n = 8 for Free-form VA, and n = 5 for P5-VA. Data are presented as mean +/- SD. Statistical significance was evaluated using two-tailed Student's *t* test. A *P* value of ≤ 0.05 is statistically significant, with * $P \leq 0.05$, ** $P \leq 0.01$, *** $P \leq 0.001$, and **** $P \leq 0.0001$.

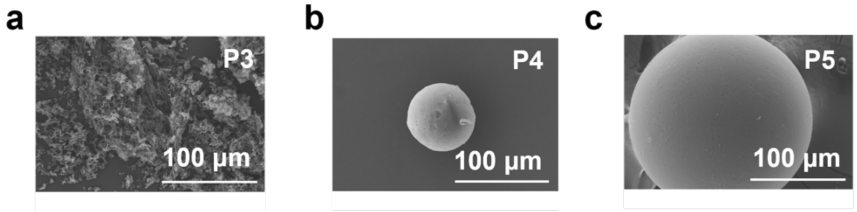


**Supplementary Figure 21. SEM images of PAE MPs with VA loading.** Morphology characterization of **(a)** P3, **(b)** P4, and **(c)** P5 MPs encapsulated with VA.

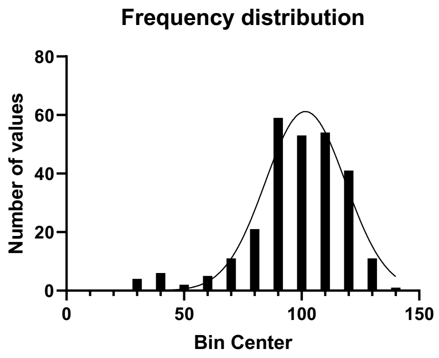


**Supplementary Figure 22. Size distribution of P5-VA MPs.** Frequency distribution of size of P5 MP with VA characterized from SEM images. The value at bin center corresponds to the diameter of MPs in the analyzed SEM images.

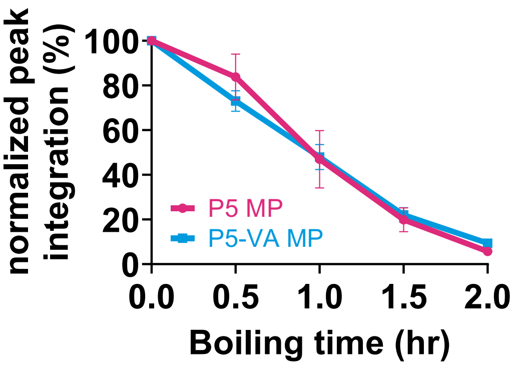


**Supplementary Figure 23. P5 polymer degradation kinetics in MP format.** Degradation kinetics of P5 MPs without cargo and P5 MPs with VA determined by NMR. n = 3 independent replicates. Data are presented as mean +/- SD.

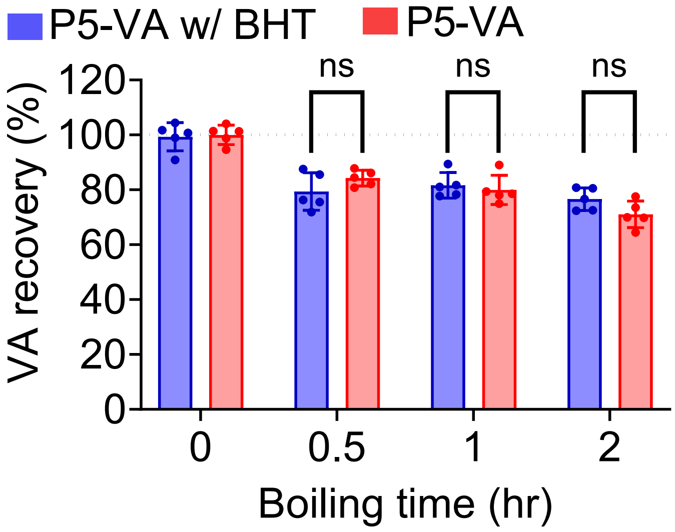


**Supplementary Figure 24. Effect of BHT on VA stabilization by P5 MPs.** No significant differences observed between P5 MPs stabilizing VA with and without BHT in boiling water treatment up to 2 hours. n = 5 independent replicates. Data are presented as mean +/- SD. Statistical significance was evaluated using two-tailed Student's *t* test. A *P* value of ≤ 0.05 is statistically significant, with * $P \leq 0.05$, ** $P \leq 0.01$, *** $P \leq 0.001$, and **** $P \leq 0.0001$.

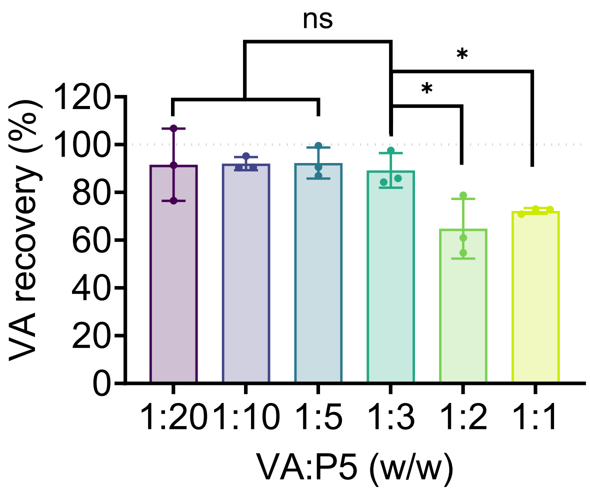


**Supplementary Figure 25. Effect of VA loading on VA stabilization by P5 MPs.** The default loading ratio was 1:10 VA:P5 (w/w). The VA recovery was studied after 2-hour water boiling treatment. The VA loading was increased to 1:3 VA:P5 (w/w) without significant decrease in VA recovery. An increase to 1:2 and 1:1 decreased the recovery of VA to close to 60%. n = 3 independent replicates. Data are presented as mean +/- SD. Statistical significance was evaluated using two-tailed Student's *t* test. A $P$ value of $\leq 0.05$ is statistically significant, with * $P \leq 0.05$, ** $P \leq 0.01$, *** $P \leq 0.001$, and **** $P \leq 0.0001$.

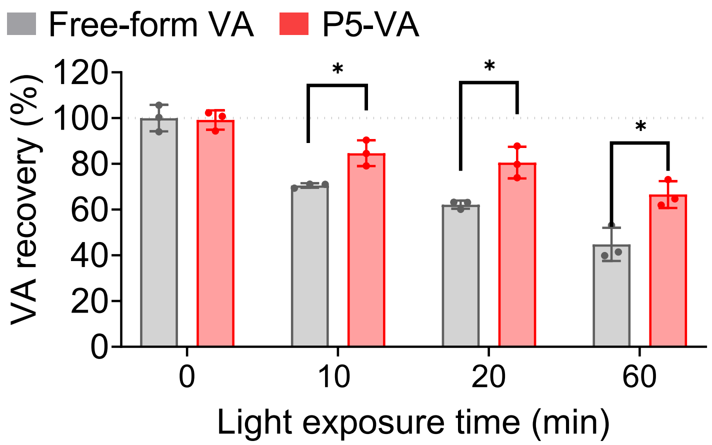


**Supplementary Figure 26. Stabilization of VA by P5 MPs under simulated sunlight.** Compared to free-form VA, use of P5 MPs enhanced VA recovery under simulated sunlight exposures. n = 3 independent replicates. Data are presented as mean +/- SD. Statistical significance was evaluated using two-tailed Student's *t* test. A *P* value of $\leq 0.05$ is statistically significant, with * $P \leq 0.05$, ** $P \leq 0.01$, *** $P \leq 0.001$, and **** $P \leq 0.0001$.

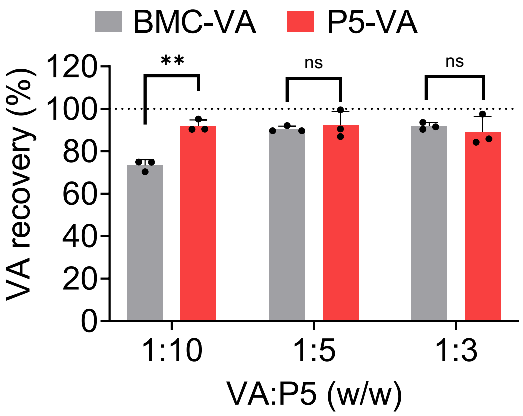


**Supplementary Figure 27. VA recovery comparison between BMC and P5 formulations.** P5 MPs provided higher or non-inferior level of protection to VA in 2-hour boiling water conditions compared to the BMC MPs. n = 3 independent replicates. Data are presented as mean +/- SD. Statistical significance was evaluated using two-tailed Student's *t* test. A *P* value of $\leq 0.05$ is statistically significant, with * $P \leq 0.05$, ** $P \leq 0.01$, *** $P \leq 0.001$, and **** $P \leq 0.0001$.

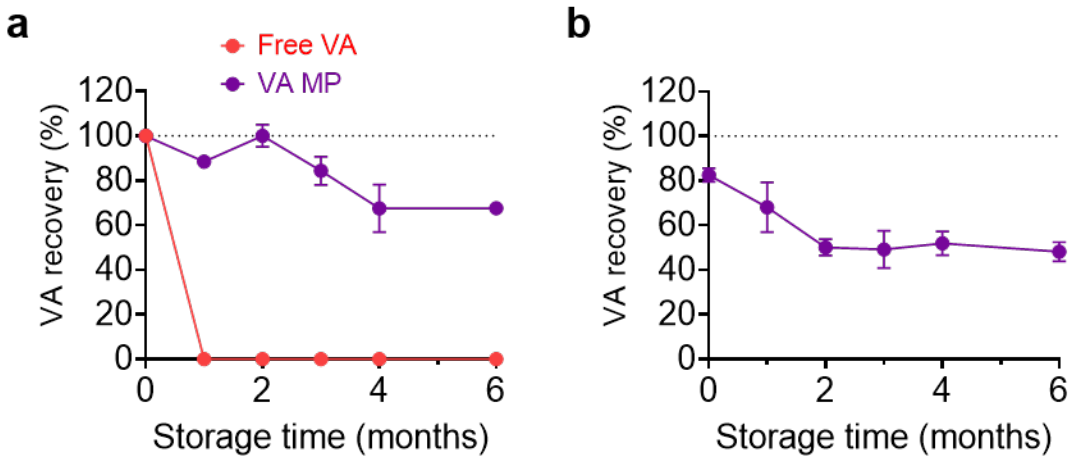


**Supplementary Figure 28. Long-term stability of VA under ambient condition.** Compared to free-form VA, P5 MPs provided effective long-term stabilization to VA under 25 °C and 40% humidity after storage **(a)** and after storage followed by 2-hour boiling in water **(b)**. n = 3 independent replicates. Data are presented as mean +/- SD.

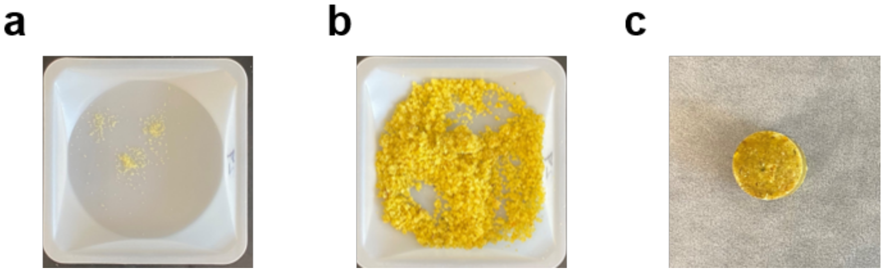


**Supplementary Figure 29. Fabrication of a bouillon cube with P5-VA MPs. (a)** A picture of P5-VA MPs placed in a weighing boat demonstrated the physical appearance of 10 mg of P5 MPs. **(b)** After mixing with ~1.5 grams of bouillon powder, the presence of P5 MPs was not recognizable by visual inspection. **(c)** The P5 MPs completely blended in the representative compressed bouillon cube.

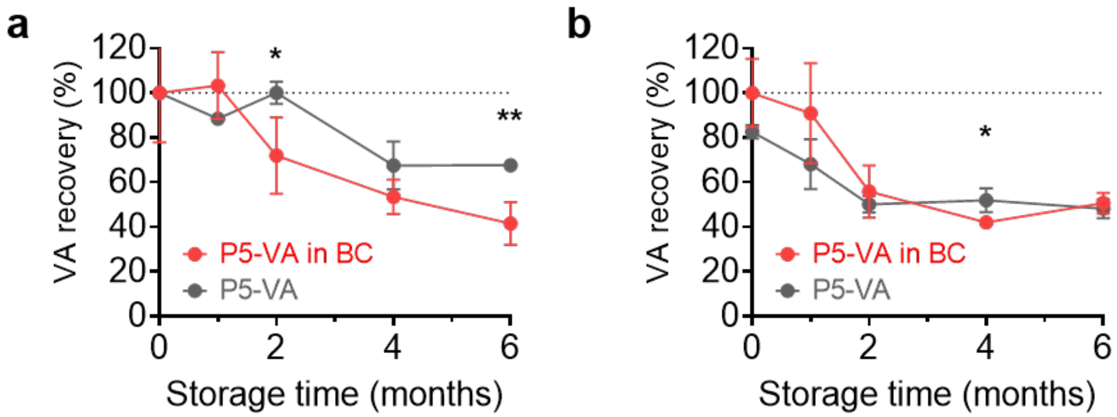


**Supplementary Figure 30. Recovery of VA from P5 MPs stored in bouillon cubes.** Stability of VA in P5-VA MPs mixed in bouillon cubes was evaluated throughout a 6-month storage time period **(a)**, and followed by 2-hour boiling in water **(b)**. The level of VA recovery was compared with P5-VA MP samples stored independently, as in without the food matrix. BC: Bouillon cube. n = 3 for P5-VA, and n = 4 for P5-VA in BC; all replicates were independent. Data are presented as mean +/- SD. Statistical significance was evaluated using two-tailed Student's *t* test. A *P* value of ≤ 0.05 is statistically significant, with * $P \leq 0.05$, ** $P \leq 0.01$, *** $P \leq 0.001$, and **** $P \leq 0.0001$.

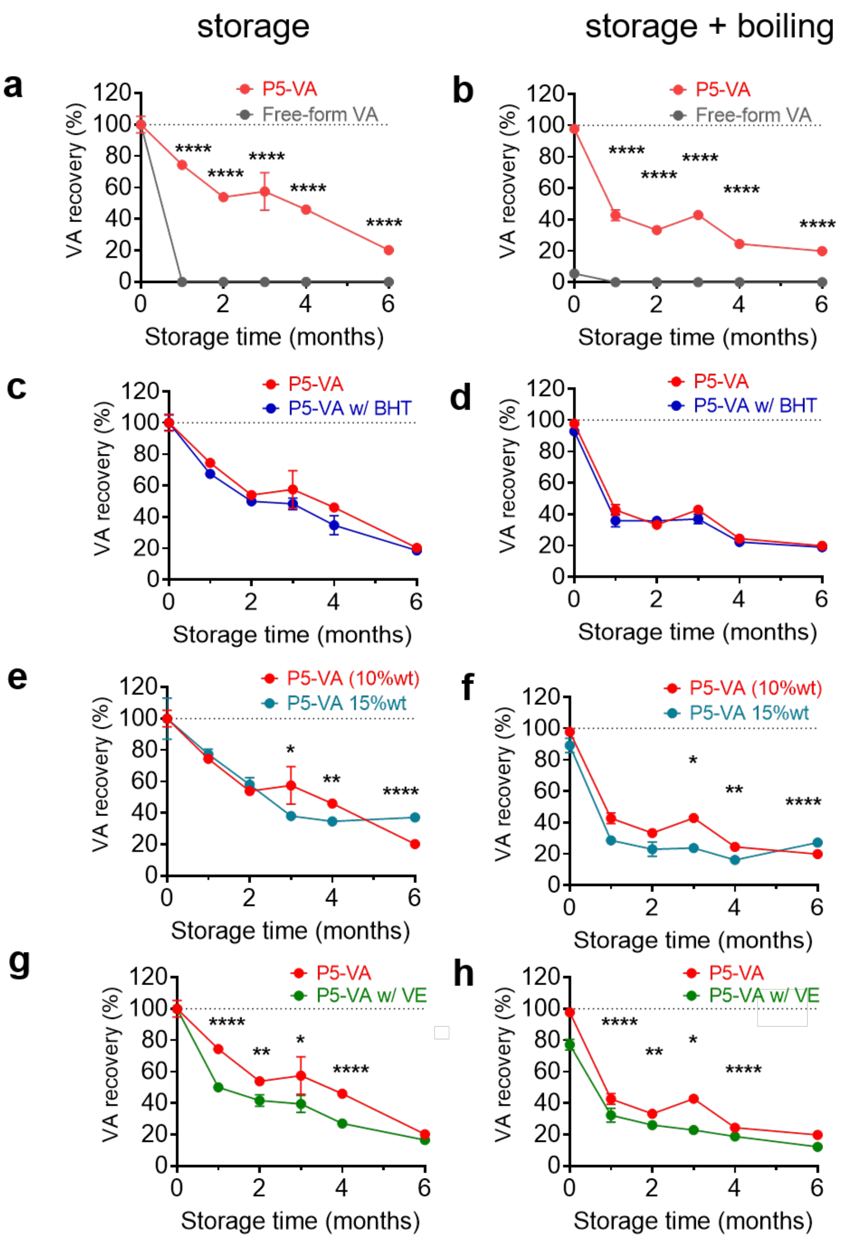


**Supplementary Figure 31. Recovery of VA from multiple P5 MPs over long-term storage under 40 °C and 75% rH and followed by extensive boiling.** Throughout 6-month storage, and for each time point followed by 2-hour water boiling treatment, VA recovery was evaluated for default P5-VA MP formulation and compared with **(a, b)** free-form VA, **(c, d)** P5-VA MPs with 0.5% BHT (w/w), **(e, f)** P5-VA MPs with VA:P5 1:7 w/w, **(g, h)** P5-VA MPs with VE, 1:1 VA:VE w/w, and 1:5 cargo:P5 w/w. n = 4 independent replicates. Data are presented as mean +/- SD. Statistical significance was evaluated using two-tailed Student's *t* test. A *P* value of $\leq 0.05$ is statistically significant, with * $P \leq 0.05$, ** $P \leq 0.01$, *** $P \leq 0.001$, and **** $P \leq 0.0001$.

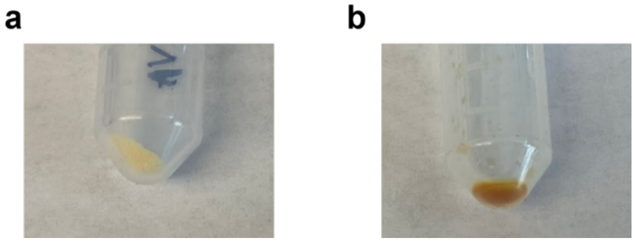

**Supplementary Figure 32. Visual comparison of stored P5 MPs.** Pictures of P5 MPs with VA stored under **(a)** RT (25 °C) and room humidity (40% rH) and **(b)** 40 °C and 75% rH at 6-month time point.

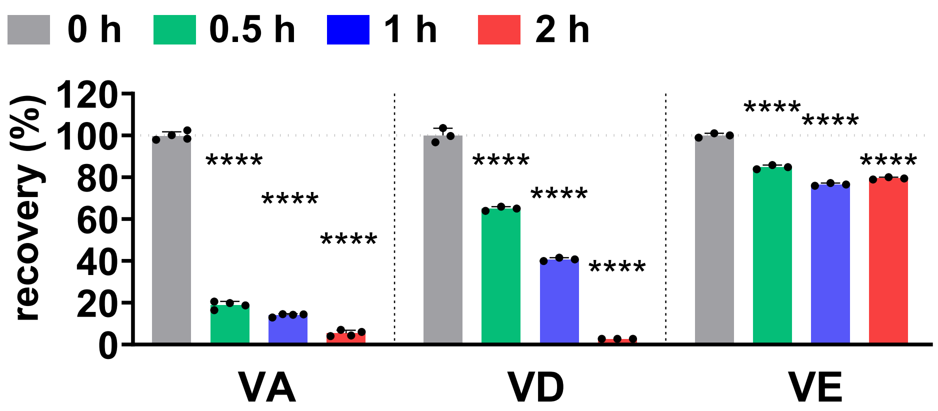


**Supplementary Figure 33. Recovery of free-form VA, VD, and VE in boiling water in time course.** VA and VD showed significant decrease in recovery in boiling, while VE showed high thermal stability. n = 4 for VA, and n = 3 for VD and VE; all replicates were independent. Data are presented as mean +/- SD. Statistical significance was evaluated using two-tailed Student's *t* test. A *P* value of ≤ 0.05 is statistically significant, with * $P \leq 0.05$, ** $P \leq 0.01$, *** $P \leq 0.001$, and **** $P \leq 0.0001$.

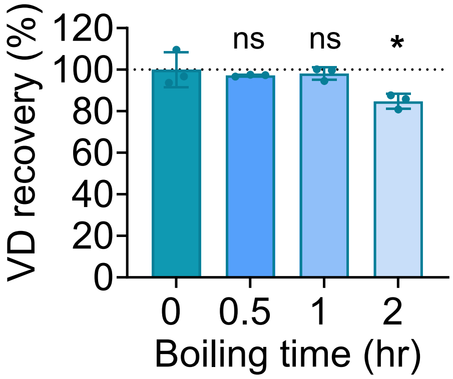


**Supplementary Figure 34. VD stabilization in P5 MPs.** Recovery of VD from P5 MP formulation in boiling water was evaluated in time course. n = 3 independent replicates. Data are presented as mean +/- SD. Statistical significance was evaluated using two-tailed Student's *t* test. A *P* value of ≤ 0.05 is statistically significant, with * *P* ≤ 0.05, ** *P* ≤ 0.01, *** *P* ≤ 0.001, and **** *P* ≤ 0.0001.

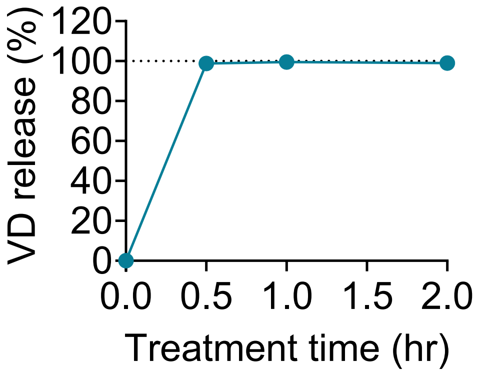


**Supplementary Figure 35. VD release from P5 MPs.** Release of VD from P5 MP formulation in SGF was evaluated in time course. n = 4 independent replicates. Data are presented as mean +/- SD.

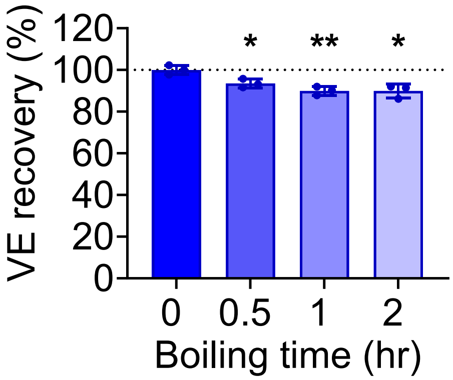


**Supplementary Figure 36. VE stabilization in P5 MPs.** Recovery of VE from P5 MP formulation in boiling water was evaluated in time course. n = 3 independent replicates. Data are presented as mean +/- SD. Statistical significance was evaluated using two-tailed Student's *t* test. A *P* value of $\leq 0.05$ is statistically significant, with * $P \leq 0.05$, ** $P \leq 0.01$, *** $P \leq 0.001$, and **** $P \leq 0.0001$.

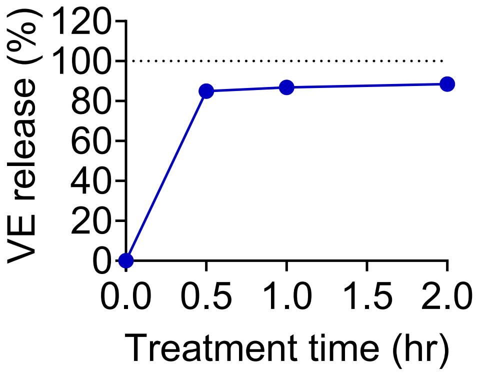


**Supplementary Figure 37. VE release from P5 MPs.** Release of VE from P5 MP formulation in SGF was evaluated in time course. n = 4 independent replicates. Data are presented as mean +/- SD. n = 4 independent replicates. Data are presented as mean +/- SD.

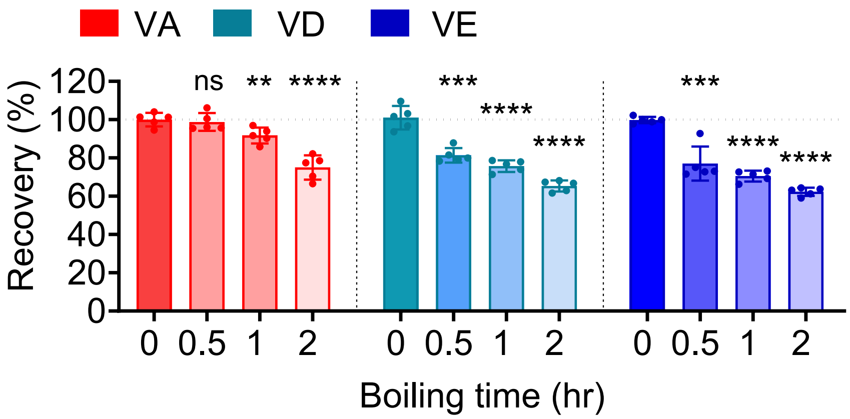


**Supplementary Figure 38. Stabilization of collectively encapsulated VA, VD, and VE in time course.** Recovery of VA, VD, and VE in boiling water from P5 MPs was evaluated in a 2-hour time period. n = 5 independent replicates. Data are presented as mean +/- SD. Statistical significance was evaluated using two-tailed Student's *t* test. A *P* value of $\leq 0.05$ is statistically significant, with * $P \leq 0.05$, ** $P \leq 0.01$, *** $P \leq 0.001$, and **** $P \leq 0.0001$.

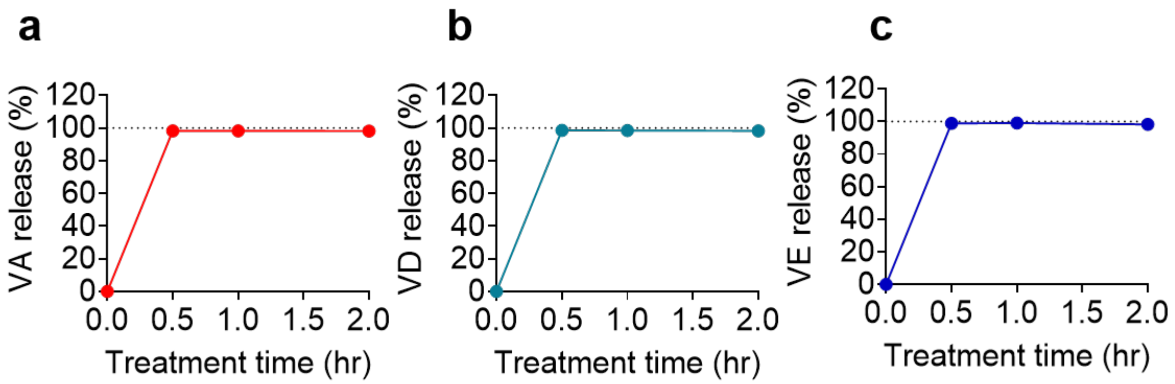


**Supplementary Figure 39. Release of collectively encapsulated VA, VD, and VE.** P5 MPs collectively encapsulated with VA, VD, and VE were treated with SGF, and release of cargo was evaluated. n = 4 independent replicates. Data are presented as mean +/- SD.

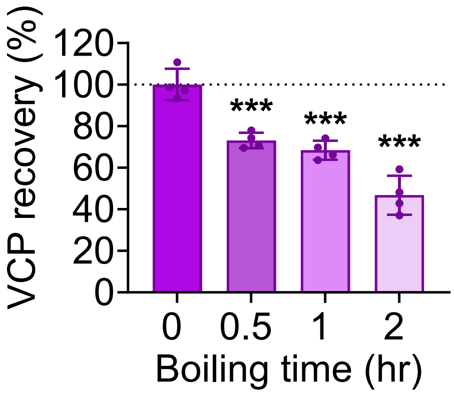


**Supplementary Figure 40. VCP stabilization in P5 MPs.** Recovery of VCP from P5 MP formulation in boiling water was evaluated in time course. n = 4 independent replicates. Data are presented as mean +/- SD. Statistical significance was evaluated using two-tailed Student's *t* test. A $P$ value of ≤ 0.05 is statistically significant, with * $P \leq 0.05$, ** $P \leq 0.01$, *** $P \leq 0.001$, and **** $P \leq 0.0001$.

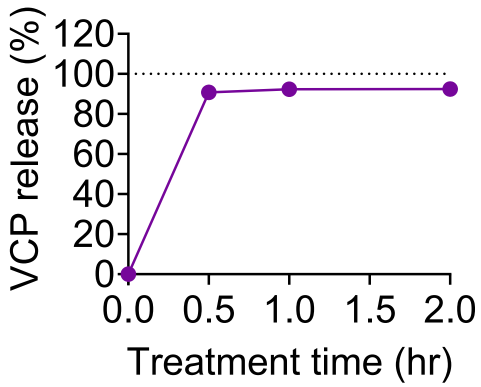


**Supplementary Figure 41. VCP release from P5 MPs.** Release of VCP from P5 MP formulation in SGF was evaluated in time course. n = 4 independent replicates. Data are presented as mean +/- SD.

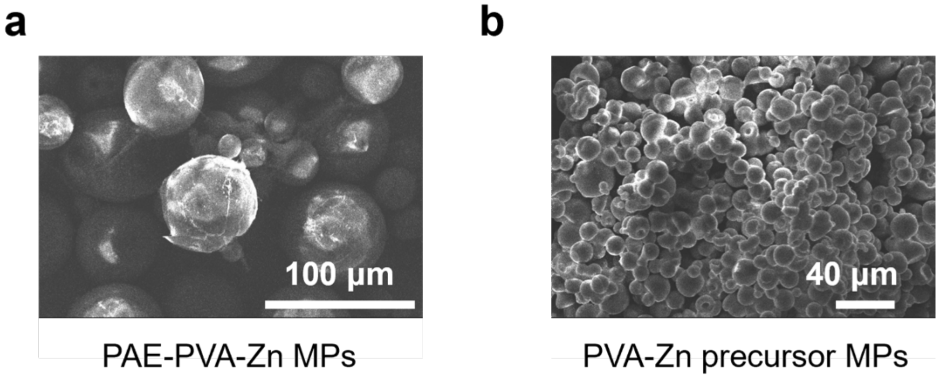


**Supplementary Figure 42. SEM images of P5 MPs formulated with Zn**. **(a)** PAE-PVA-Zn MPs and **(b)** PVA-Zn precursor MPs were characterized by SEM imaging.

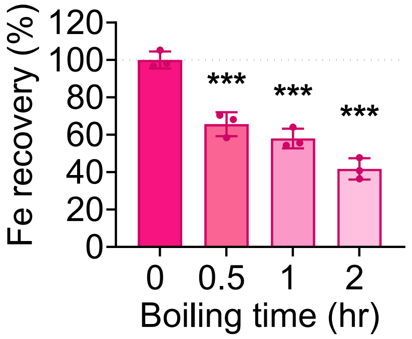


**Supplementary Figure 43. Iron stabilization in P5 MPs.** Recovery of iron from P5 MP formulation in boiling water was evaluated in time course. n = 3 independent replicates. Data are presented as mean +/- SD. Statistical significance was evaluated using two-tailed Student's *t* test. A *P* value of ≤ 0.05 is statistically significant, with * $P \leq 0.05$, ** $P \leq 0.01$, *** $P \leq 0.001$, and **** $P \leq 0.0001$.

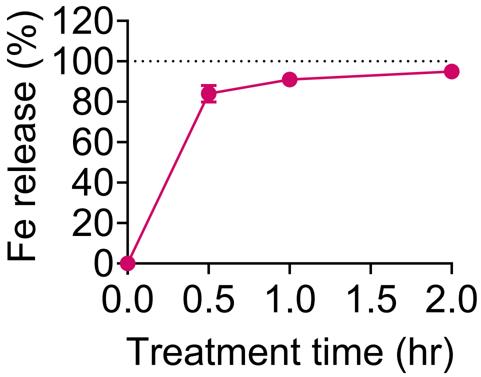


**Supplementary Figure 44. Iron release from P5 MPs.** Release of iron from P5 MP formulation in SGF was evaluated in time course. n = 3 independent replicates. Data are presented as mean +/- SD.

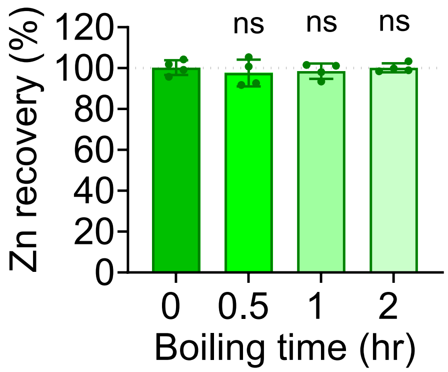


**Supplementary Figure 45. Zinc stabilization in P5 MPs.** Recovery of zinc from P5 MP formulation in boiling water was evaluated in time course. n = 4 independent replicates. Data are presented as mean +/- SD. Statistical significance was evaluated using two-tailed Student's *t* test. A *P* value of ≤ 0.05 is statistically significant, with * $P \leq 0.05$, ** $P \leq 0.01$, *** $P \leq 0.001$, and **** $P \leq 0.0001$.

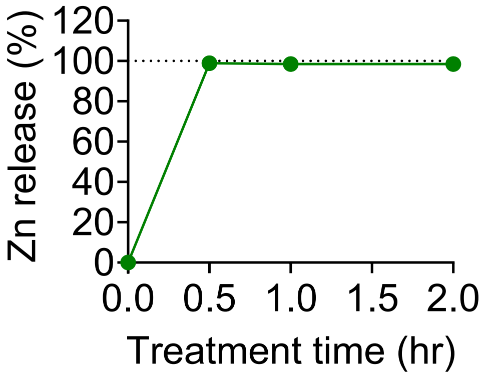


**Supplementary Figure 46. Zinc release from P5 MPs.** Release of zinc from P5 MP formulation in SGF was evaluated in time course. n = 4 independent replicates. Data are presented as mean +/- SD.

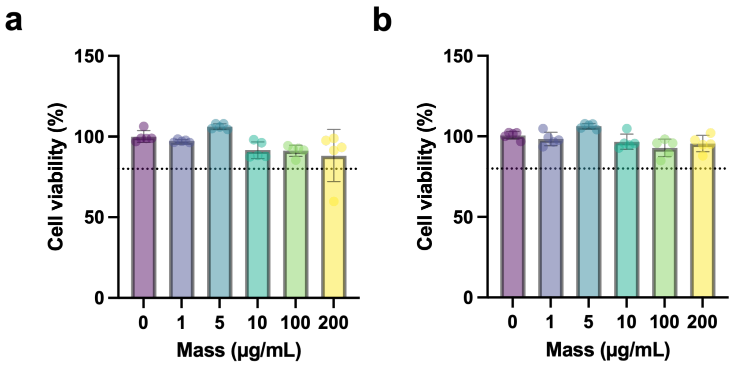


**Supplementary Figure 47. Cytotoxicity of P5 polymer and its degradation byproducts.** The cytotoxicity of raw (a) and fully degraded (b) P5 polymer was evaluated using Caco-2 cells, with no or minimal cytotoxicity observed for both test articles. n = 5 independent biological replicates. Data are presented as mean +/- SD.

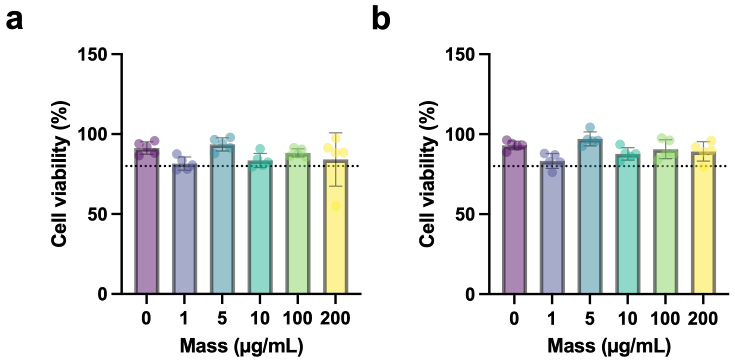


**Supplementary Figure 48. Cytotoxicity of P5-VA MPs before and after 2-hour boiling in water.** The cytotoxicity of raw (a) and 2-hour boiled (b) P5-VA MPs was assessed using Caco-2 cells, similarly with no or minimal cytotoxicity observed for both test articles. n = 5 independent biological replicates. Data are presented as mean +/- SD.

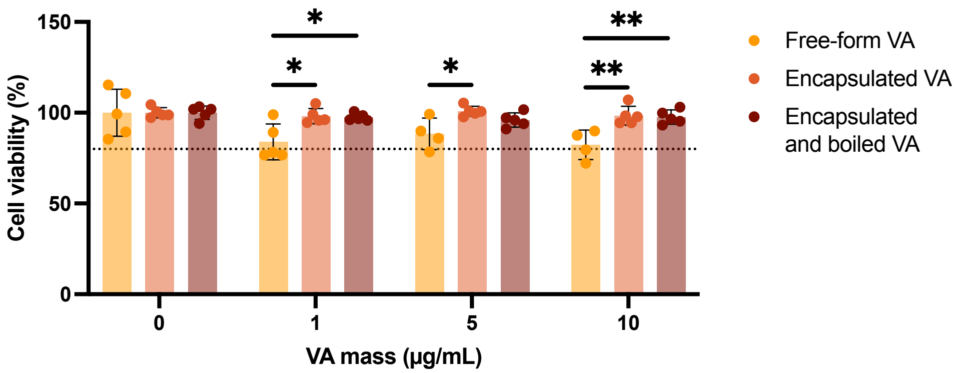


**Supplementary Figure 49. Cytotoxicity of free-form VA and P5 MP-encapsulated VA.** The cytotoxicity of free-form VA was compared with VA encapsulated by P5 MPs, including both raw MPs and those boiled in water for 2 hours. Caco-2 cells were used, with VA concentration controlled in the test articles. Minimal cytotoxicity was observed in all groups, with encapsulated VA showing relatively higher cell viability. n = 5 independent biological replicates. Data are presented as mean +/- SD. Statistical significance was evaluated using two-tailed Student's *t* test. A $P$ value of $\leq 0.05$ is statistically significant, with * $P \leq 0.05$, ** $P \leq 0.01$, *** $P \leq 0.001$, and **** $P \leq 0.0001$.

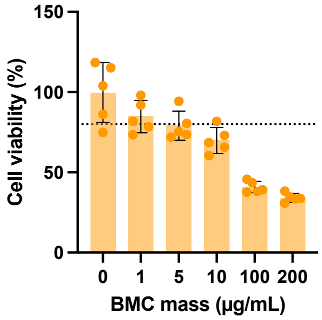


**Supplementary Figure 50. Cytotoxicity of BMC polymer.** BMC, a GRAS-status polymer for food fortification, was selected as a comparison to the P5 polymer for safety evaluation. BMC was used to treat Caco-2 cells in the same manner as the P5 polymer, with the latter showing higher cell viability. n = 5 independent biological replicates. Data are presented as mean +/- SD.

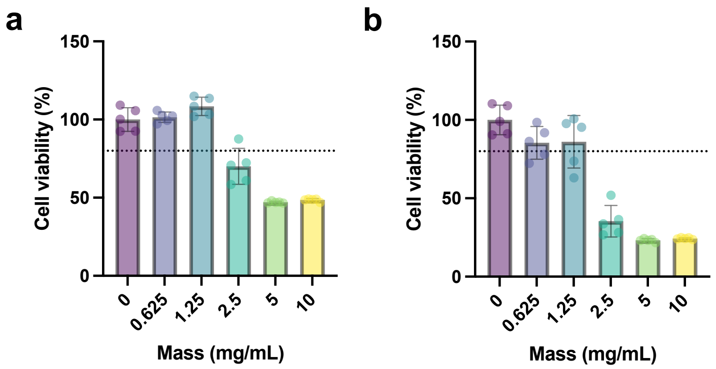


**Supplementary Figure 51. Cytotoxicity of raw P5 polymer at high concentration.** The dosage of the raw P5 polymer was increased to evaluate its cytotoxicity profile in both Caco-2 (a) and HEK-293 (b) cell lines. The P5 polymer showed minimal toxicity to both Caco-2 and HEK-293 cells up to a concentration of 1.25 mg/mL. n = 5 independent biological replicates. Data are presented as mean +/- SD.

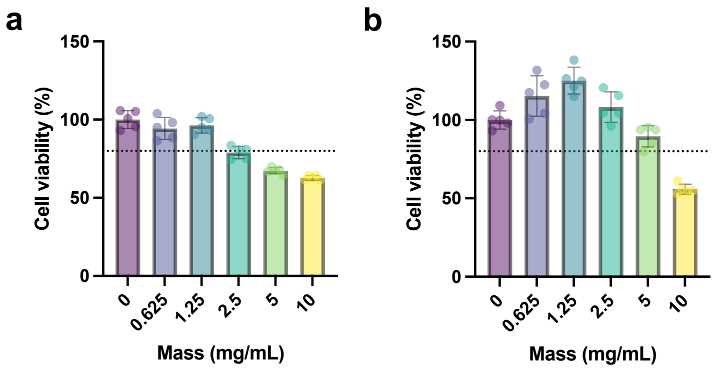


**Supplementary Figure 52. Cytotoxicity of P5 degradation byproducts at high concentration.** The dosage of the fully degraded P5 polymer was increased to evaluate its cytotoxicity profile in both Caco-2 (a) and HEK-293 (b) cell lines. The P5 polymer showed minimal toxicity to Caco-2 and HEK-293 cells up to a concentration of 2.5 mg/mL and 5 mg/mL, respectively. n = 5 independent biological replicates. Data are presented as mean +/- SD.

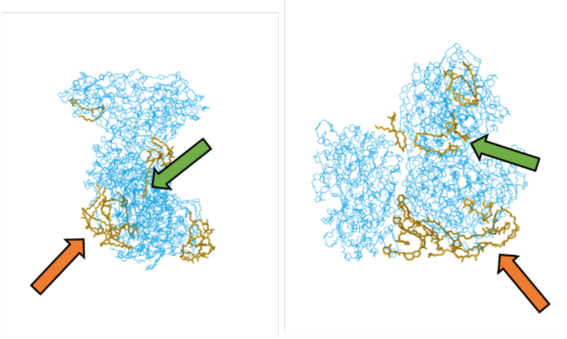

**Supplementary Figure 53. Interactions of P5 polymer and VA molecules in MD simulations.** When P5 polymer and VA molecules were placed in MD simulation, VA molecules would either adsorbed on the surface of P5 polymer globule (orange arrows) or be wrapped between P5 polymers (green arrows).

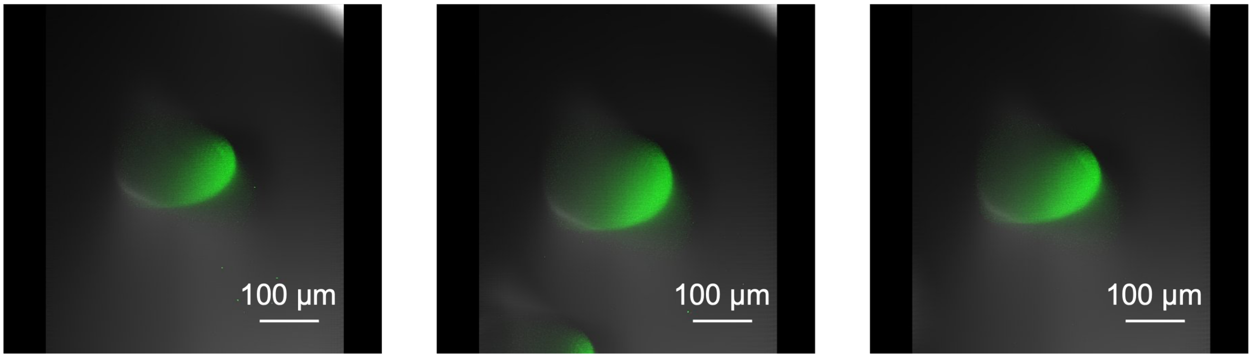


**Supplementary Figure 54. Fluorescence imaging of P5-VA MPs.** Three representative images captured by multiphoton laser scanning confocal microscope, which showed distribution of VA across P5 MPs.

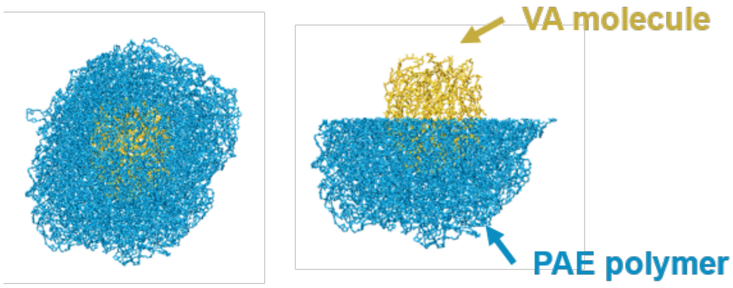


**Supplementary Figure 55. P5 polymer globule with VA core.** A P5 polymer globule with a core of VA molecules was used as the initial structure of the MD simulation runs. Water molecules not shown for clarity.

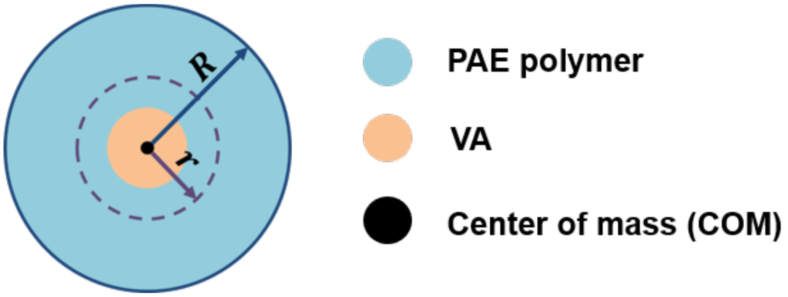


**Supplementary Figure 56. A schematic of P5 polymer globule with the VA core.** R: the normalized radius between the globule COM and the surface. r: a relative distance between the globule COM and a hypothetical inner sphere. The relative EE is the percentage of heavy atoms of VA molecules in the hypothetical inner sphere out of all heavy atoms of VA in the system. Heavy atoms are any atoms except hydrogen.

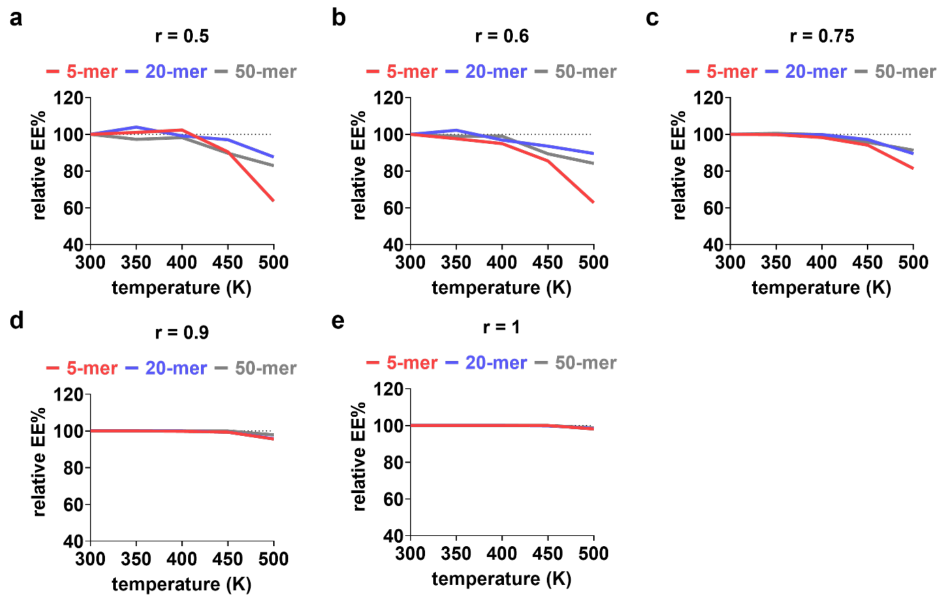


**Supplementary Figure 57. Relative EE of VA molecules at selected relative distance to COM (*r*).** The relative EE of heavy atoms of VA molecules decreased with an increase in temperature at $r$ was **(a)** 0.5, **(b)** 0.6, and **(c)** 0.75 for all cases of polymer lengths. The trend was less significant at $r$ = 0.9 **(d)** and practically vanished at $r$ = 1 **(e)**.

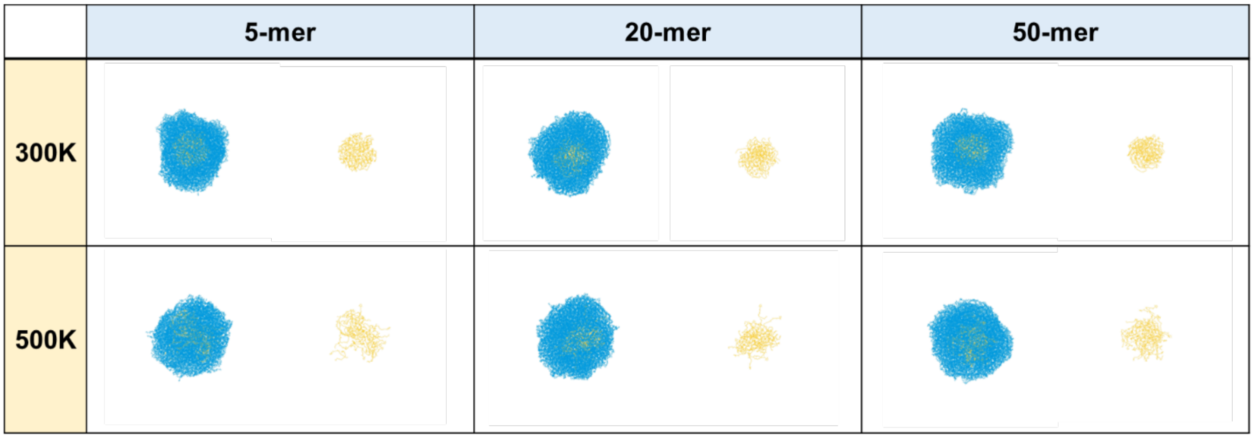


**Supplementary Figure 58. Visual presentation of P5 polymer globules with a VA core at the end of MD simulation runs.** Snapshots of the P5 polymer globules with the VA core were captured for all six simulated conditions. The VA cores were presented separated under each condition, demonstrating a higher diffusion rate of VA molecules under high-temperature conditions.

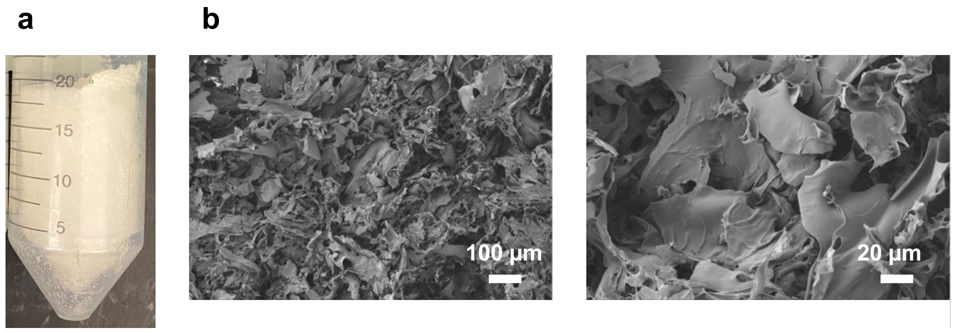


**Supplementary Figure 59. Picture and SEM images of VA formulated with P5 degradation byproducts.** P5 degradation byproducts were formulated with VA to assess whether P5 polymer had protective effect on VA when it was under high degradation. As shown in the picture (a) and SEM images (b), amorphous solid material was obtained, with no particle structure observed.

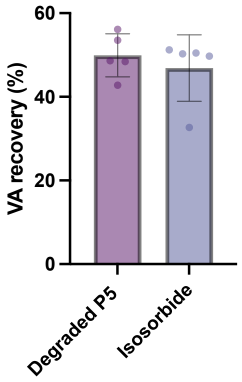


**Supplementary Figure 60. VA recovery from formulations with P5 degradation byproducts and isosorbide.** The solid materials formulated with either fully degraded P5 polymer or isosorbide, one of the degradation byproducts, were treated with 2-hour boiling in water. Both formulations showed higher VA recovery than free-form VA under the same conditions (6%). n = 5 independent replicates. Data are presented as mean +/- SD.

a b

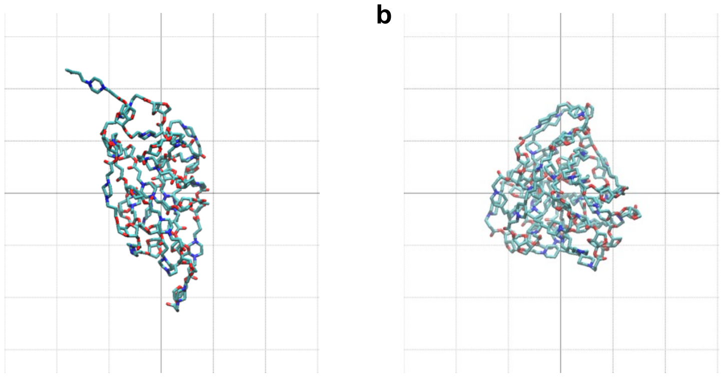

**Supplementary Figure 61. Visual comparison of MD-simulated P1 and P5 polymer globules.** One molecule of 20-mer P1 (a) and 20-mer P5 (b) polymer was simulated under the same conditions at 300K. The extended structure of the P1 polymer indicates higher mobility and solubility, corresponding to lower hydrophobicity and a reduced ability to interact with VA.

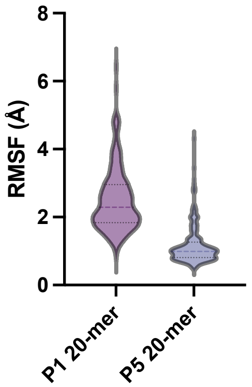


**Supplementary Figure 62. RMSF of P1 and P5 polymers.** The mobility of one molecule of 20-mer P1 and P5 polymers was quantified by RMSF. The RMSF of P1 was significantly higher than that of P5, consistent with the visual comparison of simulated molecules and experimental results of MP formation.

**Supplementary Table 1. Micronutrient loading and yield for PAE MPs.** All values are Mean +/- SD.

| Polymer | Micronutrient | Loading (µg/mg MP) | MP yield (%) |
|---|---|---|---|
| **P3** | VA | 135.1 +/- 1.5 | 61.9 +/- 8.2 |
| **P4** | VA | 95.2 +/- 2.9 | 64.6 +/- 0.6 |
| **P5** | VA | 96.5 +/- 2.8 | 59.6 +/- 3.7 |
| | VD | 94.0 +/- 8.2 | 68.6 +/- 4.2 |
| | VE | 151.3 +/- 2.5 | 68.3 +/- 7.2 |
| | VCP | 9.4 +/- 0.7 | 74.8 +/- 7.3 |
| | Iron | 4.7 +/- 0.2 | 74 +/- 14.1 |
| | Zinc | 3.3 +/- 0.2 | 68.6 +/- 9.7 |

**Supplementary Table 2. Release and recovery profile of VA in natural polymer complex formulations based on lignin.** All values are Mean +/- SD.

| Formulation | Mass ratio | Release in SGF (%) | Recovery after 2-hour boiling in water (%) |
|---|---|---|---|
| Lignin/VA | 0.1:1 | 27 +/- 4.4 | 45 +/- 12 |
| Lignin/VA | 1:1 | 9 +/- 3 | 62 +/- 2 |
| Lignin/Soy protein/VA | 0.7:0.3:1 | 57 +/- 5.5 | 34 +/- 0.3 |

**Supplementary Table 3. Associated compounds in P5 polymer synthesis.**

| Impurity type | Chemical (Method) | Method | Max Limit (ppm) |
|---|---|---|---|
| **Reagent** | Isosorbide diacrylate | HPLC | GMP |
| | TDP | CAD | GMP |
| | BHT | HPLC | 1000 |
| | Acrylic acid | HPLC | 250 |
| | TEA | GC-MS | 5000 |
| **Degradation byproducts** | Isosorbide | GC-MS | GMP |
| | di-beta-amino acid | LC-MS | GMP |
| **Solvent** | DCM | GC-MS | 600 |
| | THF | GC-MS | 720 |
| | Heptane | GC-MS | 5000 |
| **Metal element** | Arsenic | ICP-MS | 1 |
| | Cadmium | ICP-MS | 2 |
| | Lead | ICP-MS | 2 |
| | Mercury | ICP-MS | 0.1 |

HPLC: High-performance liquid chromatography

CAD: Charged Aerosol Detection

GC-MS: Gas chromatography-mass spectrometry

LC-MS: Liquid chromatography-mass spectrometry

ICP-MS: Inductively coupled plasma mass spectrometry

GMP: Good Manufacturing Practice; as low as practical for chemicals that FDA does not give specific guidance or that limit for intake of the impurity is high than EDI of the P5 polymer.

**Supplementary Table 4. Testing results of 10-gram parallel batches of the P5 polymer.**

*Batch 1*

| Molecular weight | | | |
|---|---|---|---|
| **Testing item** | **Test value** | | |
| **Mw** | 53906 g/mol | | |
| **Mn** | 18186 g/mol | | |
| **PDI** | 2.96 | | |
| **Impurity** | | | |
| **Impurity type** | **Chemical** | **Max Limit (ppm)** | **Tested value (ppm)** |
| **Reagent** | Isosorbide diacrylate | GMP | <100 |
| | TDP | GMP | <500 |
| | BHT | 1000 | <500 |
| | Acrylic acid | 250 | <100 |
| | TEA | 5000 | <100 |
| **Degradation byproducts** | Isosorbide | GMP | <100 |
| | di-beta-amino acid | GMP | 163 |
| **Solvent** | DCM | 600 | <101 |
| | THF | 720 | <100 |
| | Heptane | 5000 | 387 |
| **Metal element** | Arsenic | 1 | <0.3 |
| | Cadmium | 2 | <0.6 |
| | Lead | 2 | <0.6 |
| | Mercury | 0.1 | <0.03 |

*Batch 2*

| Molecular weight | | | |
|---|---|---|---|
| **Testing item** | **Test value** | | |
| **Mw** | 48053 g/mol | | |
| **Mn** | 14265 g/mol | | |
| **PDI** | 2.37 | | |
| **Impurity** | | | |
| **Impurity type** | **Chemical** | **Max Limit (ppm)** | **Tested value (ppm)** |
| **Reagent** | Isosorbide diacrylate | GMP | <100 |
| | TDP | GMP | <500 |
| | BHT | 1000 | <500 |
| | Acrylic acid | 250 | <100 |

| | | | |
|---|---|---|---|
| | TEA | 5000 | <100 |
| **Degradation byproducts** | Isosorbide | GMP | <100 |
| | di-beta-amino acid | GMP | 121 |
| **Solvent** | DCM | 600 | <101 |
| | THF | 720 | <100 |
| | Heptane | 5000 | 1774 |
| **Metal element** | Arsenic | 1 | <0.3 |
| | Cadmium | 2 | <0.6 |
| | Lead | 2 | <0.6 |
| | Mercury | 0.1 | <0.03 |

_Batch 3_

| **Molecular weight** | | | |
|---|---|---|---|
| **Testing item** | **Test value** | | |
| **Mw** | 46127 g/mol | | |
| **Mn** | 15436 g/mol | | |
| **PDI** | 2.99 | | |
| **Impurity** | | | |
| **Impurity type** | **Chemical** | **Max Limit (ppm)** | **Tested value (ppm)** |
| **Reagent** | Isosorbide diacrylate | GMP | <100 |
| | TDP | GMP | <500 |
| | BHT | 1000 | <500 |
| | Acrylic acid | 250 | <100 |
| | TEA | 5000 | <100 |
| **Degradation byproducts** | Isosorbide | GMP | <100 |
| | di-beta-amino acid | GMP | 190 |
| **Solvent** | DCM | 600 | <101 |
| | THF | 720 | <100 |
| | Heptane | 5000 | 643 |
| **Metal element** | Arsenic | 1 | <0.3 |
| | Cadmium | 2 | <0.6 |
| | Lead | 2 | <0.6 |
| | Mercury | 0.1 | <0.03 |

**Supplementary Table 5. Testing results of 100-gram batch of the P5 polymer.**

| Molecular weight | | | |
|---|---|---|---|
| **Testing item** | **Test value** | | |
| **Mw** | 45389 g/mol | | |
| **Mn** | 14916 g/mol | | |
| **PDI** | 3.04 | | |
| **Impurity** | | | |
| **Impurity type** | **Chemical** | **Max Limit (ppm)** | **Tested value (ppm)** |
| **Reagent** | Isosorbide diacrylate | GMP | <100 |
| | TDP | GMP | 217 |
| | BHT | 1000 | 582 |
| | Acrylic acid | 250 | <100 |
| | TEA | 5000 | <100 |
| **Degradation byproducts** | Isosorbide | GMP | <100 |
| | di-beta-amino acid | GMP | <100 |
| **Solvent** | DCM | 600 | <101 |
| | THF | 720 | <100 |
| | Heptane | 5000 | <100 |
| **Metal element** | Arsenic | 1 | <0.3 |
| | Cadmium | 2 | <0.6 |
| | Lead | 2 | <0.6 |
| | Mercury | 0.1 | <0.03 |

**Supplementary Table 6. Number of P5 and VA molecules used in the MD simulation studies.**

| P5 polymer length | Number of P5 polymer molecules | Number of VA molecules |
|---|---|---|
| 5-mer | 150 | 150 |
| 20-mer | 38 | 150 |
| 50-mer | 15 | 150 |

## NMR Spectra

## $^{1}H$ (top) and $^{13}C$ (bottom)

Isosorbide diacrylate

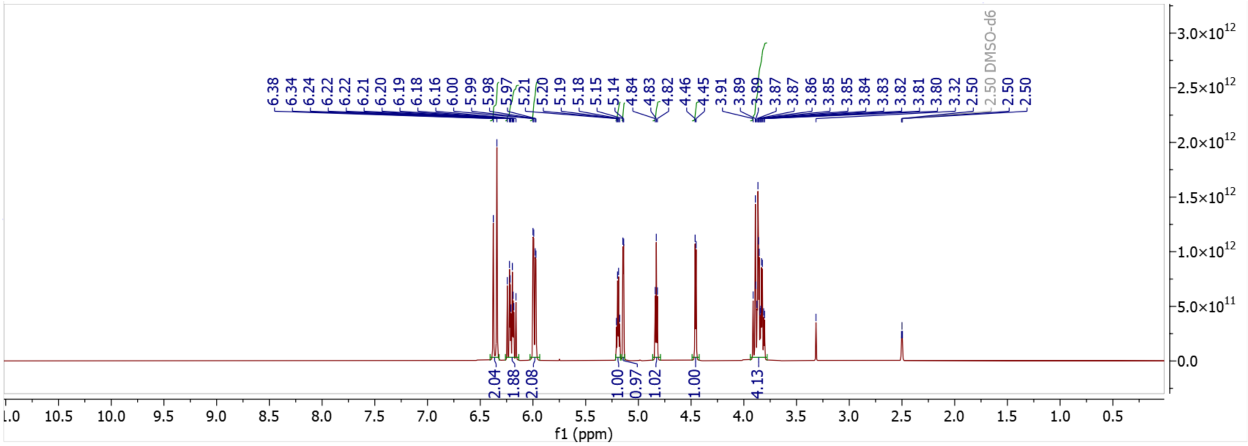


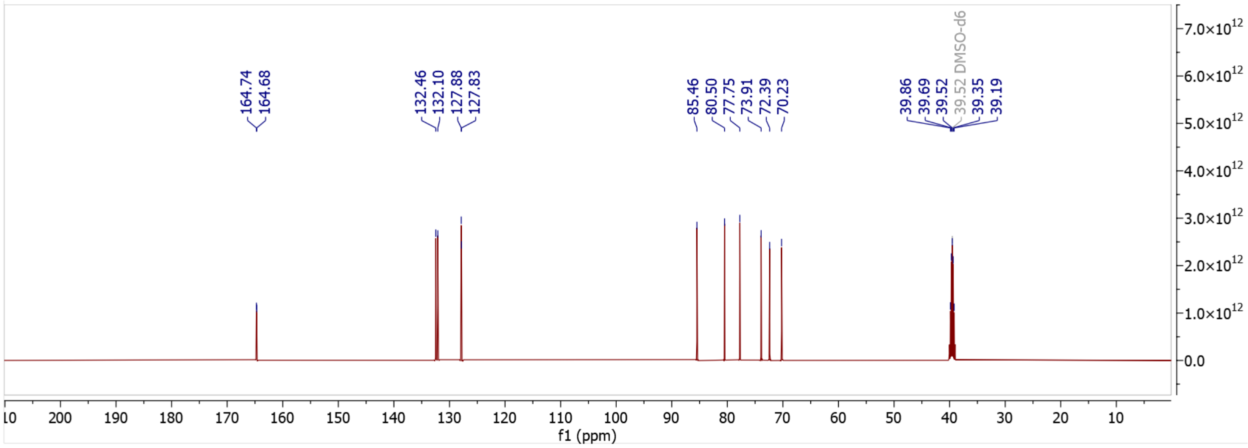

P1 polymer

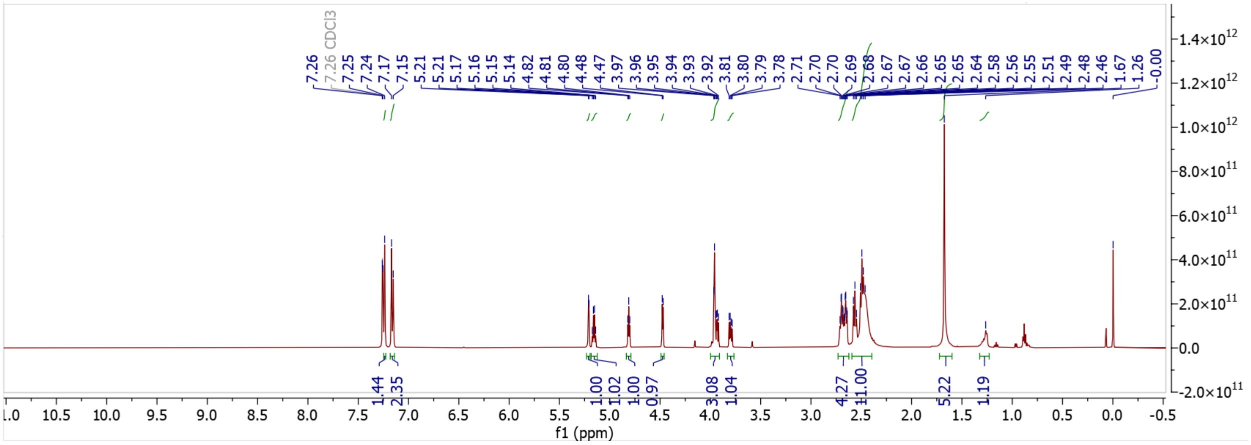

7.26
7.26 CDCl3
7.25
7.24
7.17
7.15
5.21
5.21
5.17
5.16
5.15
5.14
4.82
4.81
4.80
4.48
4.47
3.97
3.96
3.95
3.94
3.93
3.92
3.81
3.80
3.79
3.78
2.71
2.70
2.70
2.69
2.68
2.67
2.67
2.66
2.65
2.65
2.64
2.58
2.56
2.55
2.51
2.49
2.48
2.46
1.67
1.26
-0.00
1.44
2.35
1.00
1.02
1.00
0.97
3.08
1.04
4.27
11.00
5.22
1.19
f1 (ppm)


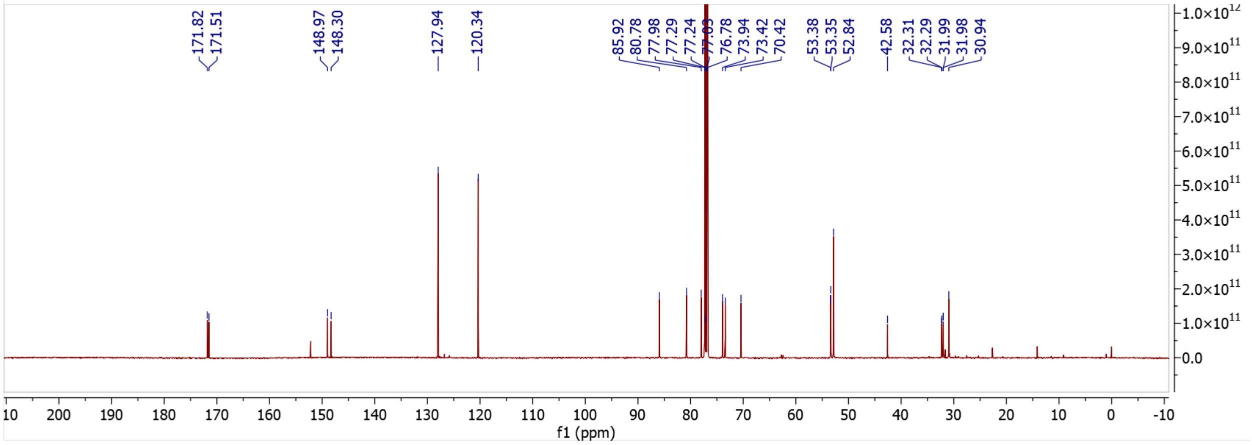

171.82
171.51
148.97
148.30
127.94
120.34
85.92
80.78
77.98
77.29
77.24
77.03
76.78
73.94
73.42
70.42
53.38
53.35
52.84
42.58
32.31
32.29
31.99
31.98
30.94
f1 (ppm)

P2 polymer

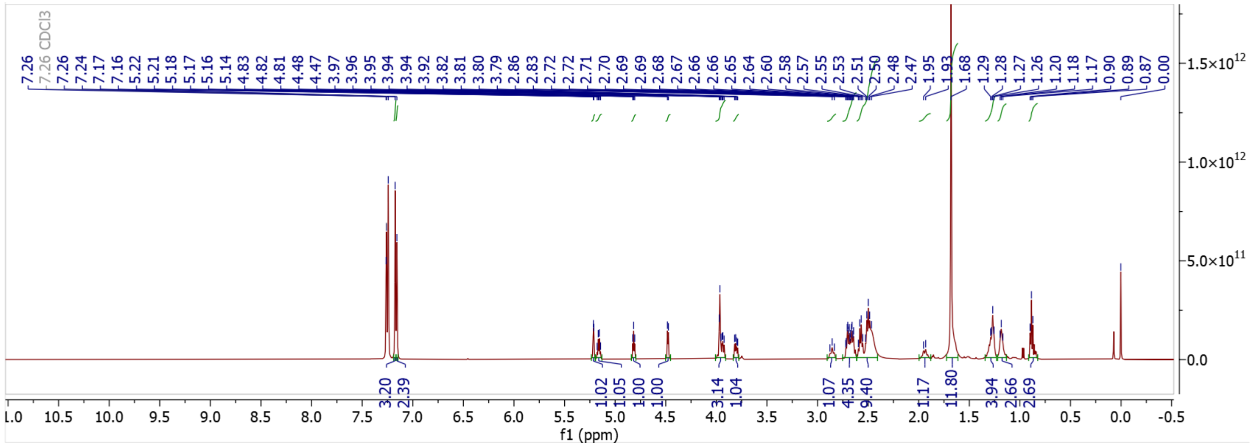
7.26
7.26 CDCl3
7.26
7.24
7.17
7.16
5.22
5.21
5.18
5.17
5.16
5.14
4.83
4.82
4.81
4.48
4.47
3.97
3.96
3.95
3.94
3.94
3.92
3.82
3.81
3.80
3.79
2.86
2.83
2.72
2.71
2.70
2.69
2.69
2.68
2.67
2.66
2.66
2.65
2.64
2.60
2.58
2.57
2.55
2.53
2.51
2.50
2.48
2.47
1.95
1.93
1.68
1.29
1.28
1.27
1.26
1.20
1.18
1.17
0.90
0.89
0.87
0.00
3.20
2.39
1.02
1.05
1.00
1.00
3.14
1.04
1.07
4.35
9.40
1.17
11.80
3.94
2.66
2.69
f1 (ppm)

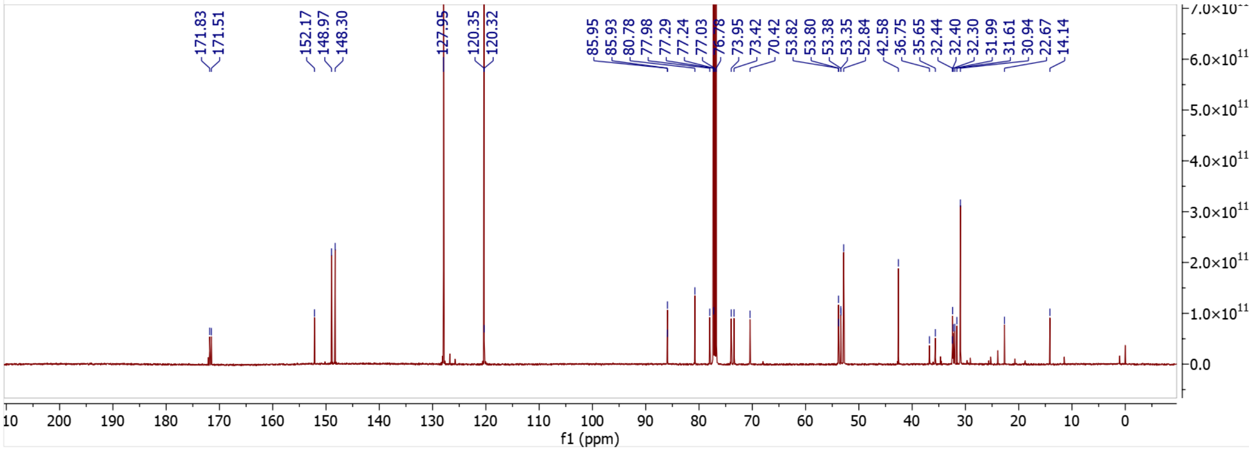
171.83
171.51
152.17
148.97
148.30
127.95
120.35
120.32
85.95
85.93
80.78
77.98
77.29
77.24
77.03
76.78
73.95
73.42
70.42
53.82
53.80
53.38
53.35
52.84
42.58
36.75
35.65
32.44
32.40
32.30
31.99
31.61
30.94
22.67
14.14
f1 (ppm)

P3 polymer

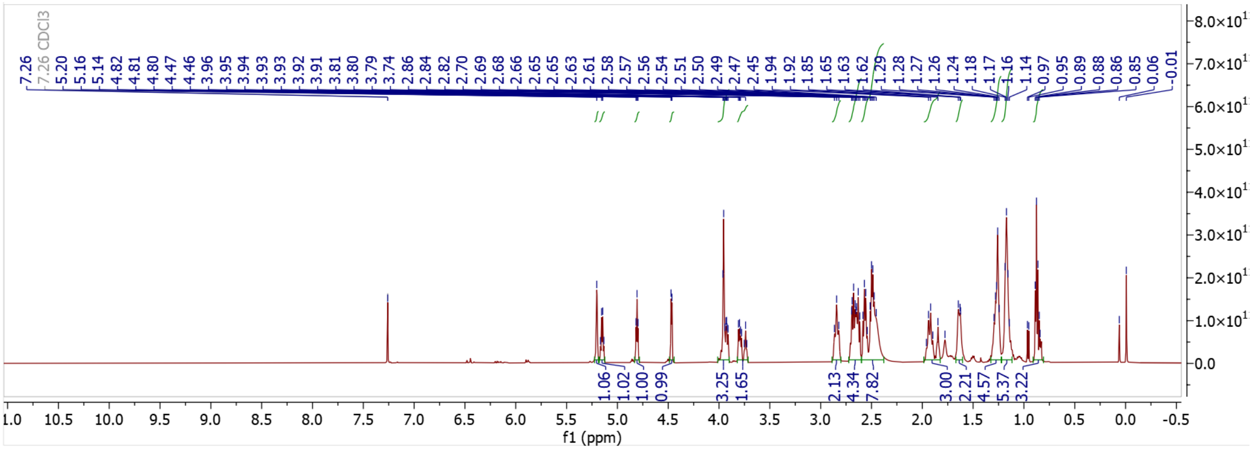


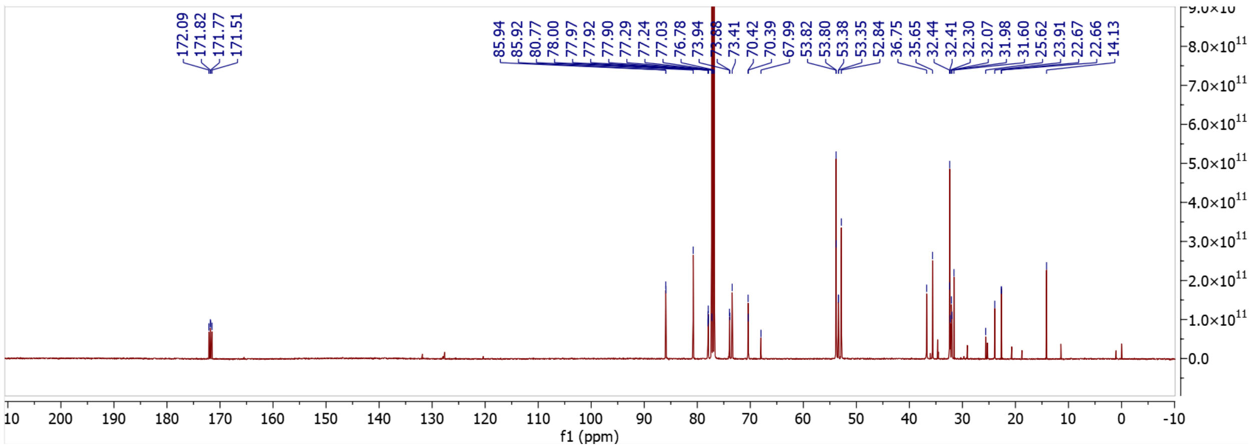

P4 polymer

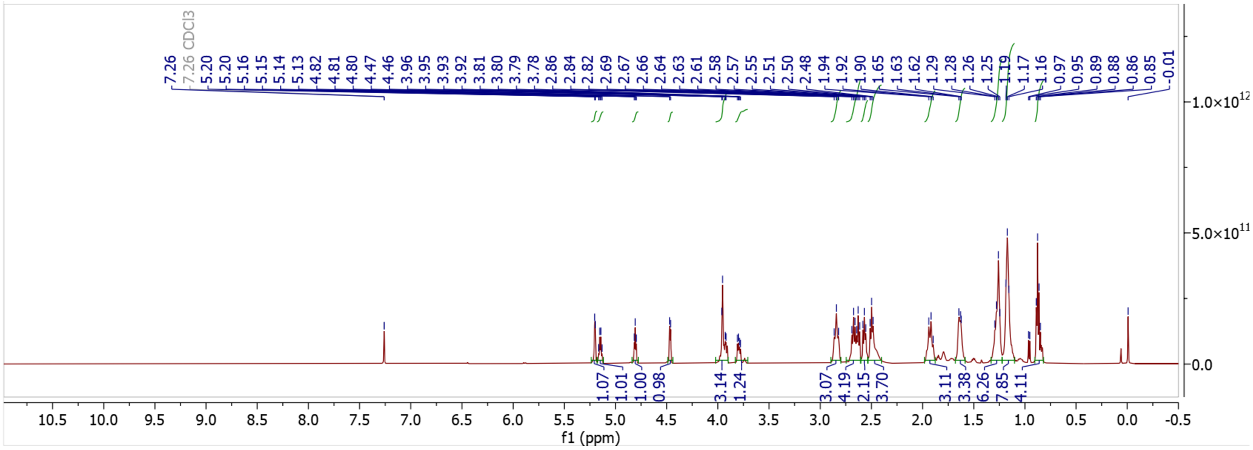

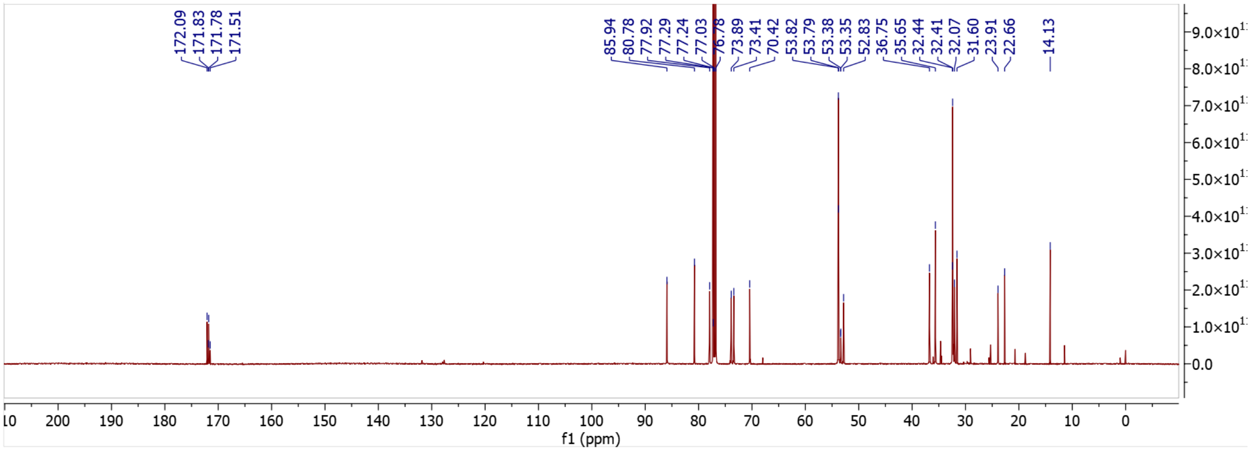

P5 polymer

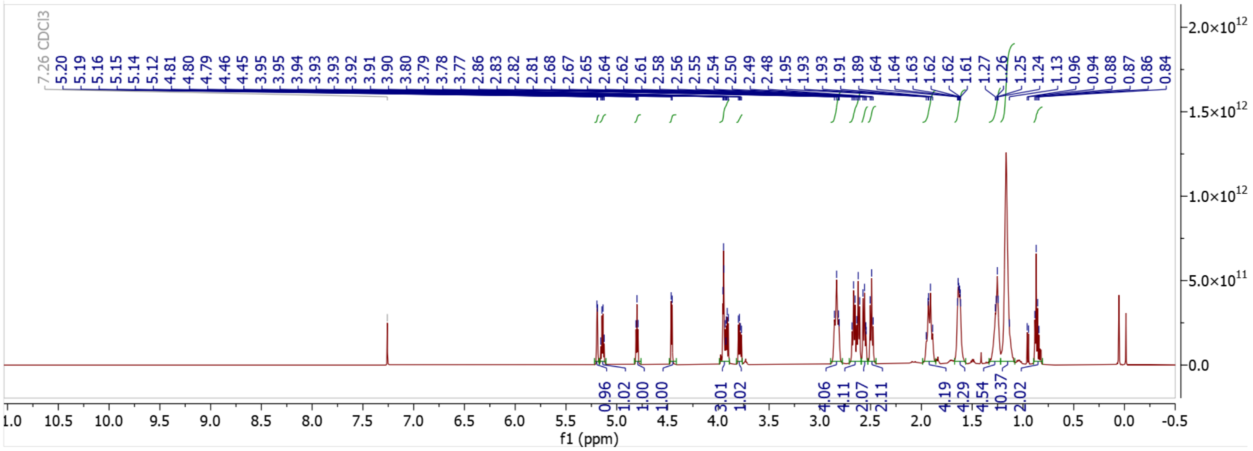
7.26 CDCl3
5.20
5.19
5.16
5.15
5.14
5.12
4.81
4.80
4.79
4.46
4.45
3.95
3.95
3.94
3.93
3.93
3.92
3.91
3.90
3.80
3.79
3.78
3.77
2.86
2.83
2.82
2.81
2.68
2.67
2.65
2.64
2.62
2.61
2.58
2.56
2.55
2.54
2.50
2.49
2.48
1.95
1.93
1.91
1.89
1.64
1.64
1.63
1.62
1.62
1.61
1.27
1.26
1.25
1.24
1.13
0.96
0.94
0.88
0.87
0.86
0.84
0.96
1.02
1.00
1.00
3.01
1.02
4.06
4.11
2.07
2.11
4.19
4.29
4.54
10.37
2.02
f1 (ppm)

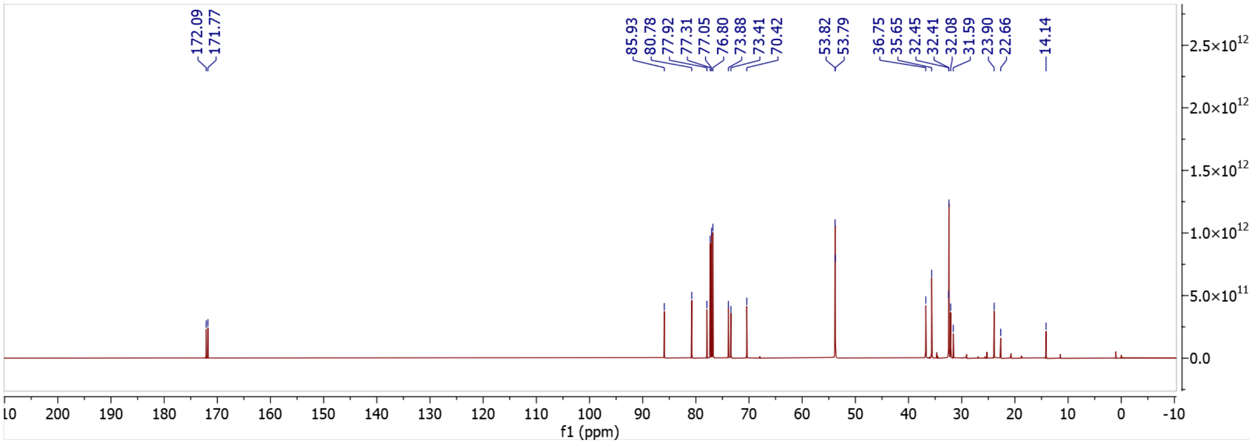
172.09
171.77
85.93
80.78
77.92
77.31
77.05
76.80
73.88
73.41
70.42
53.82
53.79
36.75
35.65
32.45
32.41
32.08
31.59
23.90
22.66
14.14
f1 (ppm)

## GPC Curves

P2 polymer

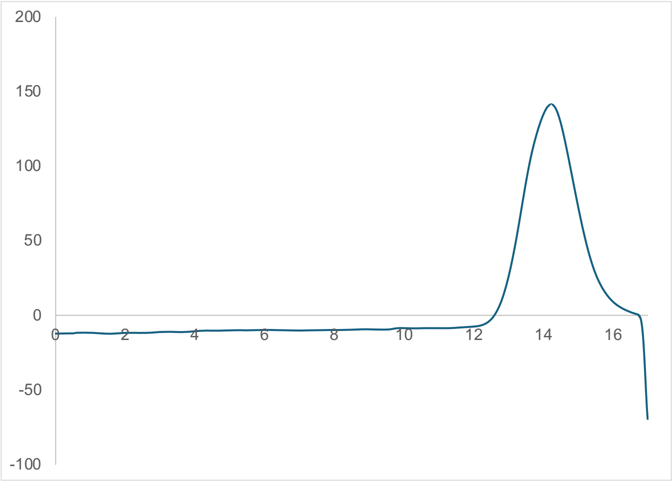


P3 polymer

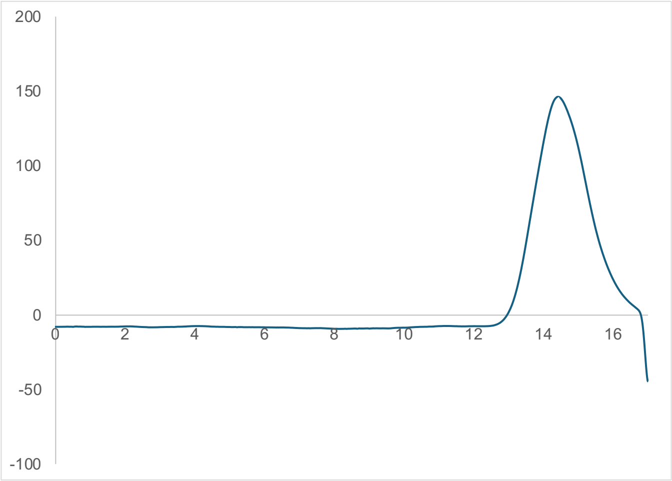

P4 polymer

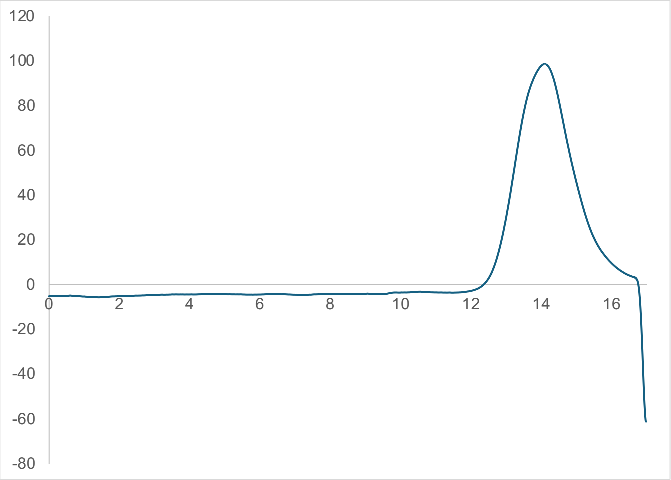


P5 polymer

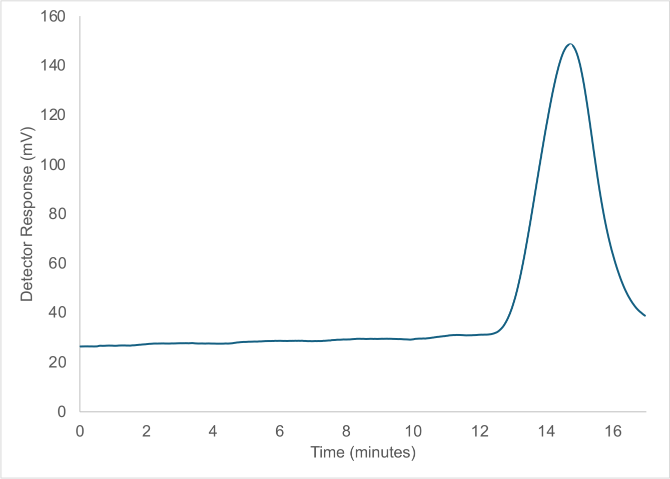

## Reference


1. Fransen, K. A. *et al.* High-throughput experimentation for discovery of biodegradable polyesters. *Proc. Natl. Acad. Sci.* **120**, e2220021120 (2023).
2. Tang, W. *et al.* Enhanced stability and clinical absorption of a form of encapsulated vitamin A for food fortification. *Proc. Natl. Acad. Sci.* **119**, e2211534119 (2022).
3. Anselmo, A. C. *et al.* A heat-stable microparticle platform for oral micronutrient delivery. *Sci. Transl. Med.* **11**, eaaw3680 (2019).
4. Nam, H. C. & Park, W. H. Aliphatic Polyester-Based Biodegradable Microbeads for Sustainable Cosmetics. *ACS Biomater. Sci. Eng.* **6**, 2440–2449 (2020).
